# The pan HDAC inhibitor Givinostat improves muscle function and histological parameters in two Duchenne muscular dystrophy murine models expressing different haplotypes of the *LTBP4* gene

**DOI:** 10.1186/s13395-021-00273-6

**Published:** 2021-07-22

**Authors:** Simonetta Andrea Licandro, Luca Crippa, Roberta Pomarico, Raffaella Perego, Gianluca Fossati, Flavio Leoni, Christian Steinkühler

**Affiliations:** 1 New Drug Incubator, Italfarmaco S.p.A., Milan, Italy; 2 University of Milano-Bicocca, Milan, Italy; 3 School of Medicine and Surgery, University of Milano-Bicocca, Milan, Italy; 4 Preclinical Development, Italfarmaco S.p.A., Milan, Italy

**Keywords:** Duchenne nuscular dystrophy, Givinostat, *mdx*, D2.B10, *LTBP4*, HDAC inhibitor

## Abstract

**Background:**

In the search of genetic determinants of Duchenne muscular dystrophy (DMD) severity, *LTBP4*, a member of the latent TGF-β binding protein family, emerged as an important predictor of functional outcome trajectories in mice and humans. Nonsynonymous single-nucleotide polymorphisms in *LTBP4* gene associate with prolonged ambulation in DMD patients, whereas an in-frame insertion polymorphism in the mouse *LTBP4* locus modulates disease severity in mice by altering proteolytic stability of the Ltbp4 protein and release of transforming growth factor-β (TGF-β). Givinostat, a pan-histone deacetylase inhibitor currently in phase III clinical trials for DMD treatment, significantly reduces fibrosis in muscle tissue and promotes the increase of the cross-sectional area (CSA) of muscles in *mdx* mice. In this study, we investigated the activity of Givinostat in *mdx* and in D2.B10 mice, two mouse models expressing different Ltbp4 variants and developing mild or more severe disease as a function of Ltbp4 polymorphism.

**Methods:**

Givinostat and steroids were administrated for 15 weeks in both DMD murine models and their efficacy was evaluated by grip strength and run to exhaustion functional tests. Histological examinations of skeletal muscles were also performed to assess the percentage of fibrotic area and CSA increase.

**Results:**

Givinostat treatment increased maximal normalized strength to levels that were comparable to those of healthy mice in both DMD models. The effect of Givinostat in both grip strength and exhaustion tests was dose-dependent in both strains, and in D2.B10 mice, Givinostat outperformed steroids at its highest dose. The in vivo treatment with Givinostat was effective in improving muscle morphology in both *mdx* and D2.B10 mice by reducing fibrosis.

**Conclusion:**

Our study provides evidence that Givinostat has a significant effect in ameliorating both muscle function and histological parameters in *mdx* and D2.B10 murine models suggesting a potential benefit also for patients with a poor prognosis *LTBP4* genotype.

**Supplementary Information:**

The online version contains supplementary material available at 10.1186/s13395-021-00273-6.

## Background

Duchenne muscular dystrophy (DMD) is a lethal X-linked disorder that leads to muscle wasting with an average incidence of 1:3500 newborn males. DMD is due to mutations in the gene encoding dystrophin, which cause the absence of the protein. The lack of dystrophin leads to muscle fiber degeneration, activation of chronic inflammatory pathways and progressive muscle tissue replacement by fibroblasts and adipocytes, triggering the process leading to fibrosis development [1]. In DMD muscles, the normal regenerative pathways are subverted and the abnormal substitution of damaged muscle fibers by fibrotic and adipose tissue leads to a severe reduction in muscle function. The main causes of the reduced life span of DMD patients are severe respiratory insufficiency, due to the weakening of the diaphragm (DIA), and cardiac failure [2].

To date there is no standard therapy for DMD patients that leads to the healing of the disease; however, glucocorticoid (GC) steroid treatment, corrective orthopedic surgery, and assisted ventilation can contribute to improve the quality of life of patients and to delay disease progression [3]. Attempts to specifically target individual mutations have recently led to the approval of exon skipping oligonucleotides (Eteplirsen, Golodirsen, and Vitolarsen) by the FDA [4–6] and Ataluren for patients with nonsense mutations by EMA [7, 8]. Only a minority of patients can benefit from these treatments, and the mainstay of disease management is GC steroids. GC steroid treatment (mainly prednisone and Deflazacort) was shown to beneficially influence all disease trajectories, including survival and prolonged ambulation in DMD patients [9–11], but this benefit comes at the cost of significant side effects such as body weight (BW) increase, growth stunting, Cushing-like symptoms, mood changes, increased incidence of fractures, and susceptibility to infections [12, 13]. Paradoxically, chronic GC steroid administration may promote muscle atrophy, and *mdx* mice subjected to chronic GC steroid treatment show a significant impairment of heart function and an increase of cardiac fibrosis, suggesting that prolonged steroid treatment may be detrimental to dystrophic heart muscle [14, 15]. Thus, there is a clear need for a broadly active and well-tolerated treatment for DMD patients.

Recently, in the search of genetic determinants of DMD severity, the *LTBP4* gene emerged as an important predictor of functional outcome trajectories. The *LTBP4* gene encodes the latent transforming growth factor-β (TGF-β)-binding protein (Ltbp) 4 that binds TGF-β in the extracellular matrix, sequestering this cytokine [16–18]. During inflammatory processes, TGF-β is released from the Ltbp4 complex by proteolysis of the proline-rich hinge domain. This cleavage leads to the activation of TGF-β [19]. Once liberated from the complex, free TGF-β regulates collagen deposition and promotes fibrotic processes [20]. During the physiological healing process, a transient release of TGF-β is necessary, whereas sustained pro-inflammatory cytokine levels contribute to the pathological tissue degeneration processes, such as fibrosis [21]. High TGF-β levels have been shown to correlate with the severity of muscle fibrosis in DMD [21].

In humans, single-nucleotide polymorphisms (SNPs) have been identified in the Ltbp4 protein, which originate VTTT and IAAM haplotypes [17]. DMD patients homozygous for the IAAM Ltbp4 haplotype remained ambulatory significantly longer (12.5 ± 3.3 years) than those homozygous for the VTTT haplotype (10.7 ± 2.1 years) [17]. In addition, IAAM fibroblasts exposed to TGF-β show a reduction in phospho-SMAD levels when compared to VTTT fibroblasts, in line with the concept that *LTBP4* regulates TGF-β activity [17, 22, 23].

There are two mouse strains that mimic the two different human Ltbp4 haplotypes: C57BL10ScSn-Dmd^*mdx*^/J (hereafter referred to as *mdx*) have a 12-amino-acid insertion in the proline-rich region of Ltbp4. This contributes to a mild DMD phenotype due to a lower sensitivity to proteolysis and a reduced activation of TGF-β as it occurs in the human IAAM haplotype [16]; D2.B10-Dmd^*mdx*^/J (hereafter referred to as D2.B10) have a 12-amino-acid deletion in the same Ltbp4 region and functionally resemble the human VTTT haplotype (severe DMD phenotype due to increased sensitivity to proteolysis and increased TGF-β activity) [16]. *Mdx* mice harbor a spontaneous point mutation in exon 23 of the dystrophin gene, leading to the loss of dystrophin [24] and are routinely used as a rodent model of the DMD disease even though it has a milder phenotype as compared to DMD patients and a normal lifespan. The comparatively mild phenotype of *mdx* mice can also be attributed to the compensatory function of the dystrophin-related protein utrophin, which is highly upregulated in regenerating muscle fibers in adult *mdx* mutants, except for the DIA muscle [24]. The D2.B10 strain, created by backcrossing *mdx* mice onto the DBA/2J background, may be a superior DMD model as it better recapitulates several of the human characteristics of the DMD myopathy (reduced hindlimb muscle weight, fewer and atrophied myofibers, increased fibrosis, fat deposition and inflammatory infiltrate, muscle weakness) when compared to strains with this mutant allele on other genetic backgrounds (such as *mdx* mice) [24–26].

Pharmacological blockade of histone deacetylases (HDACs) decreases fibrosis and promotes compensatory regeneration in the *mdx* skeletal muscle [27–30]. The role of HDACs in the DMD muscle is not fully understood; however, if comparing the muscles of *mdx* mice to that of healthy C57BL/10 J mice, it can be observed that the HDAC activity is high in *mdx* mice [28]. Givinostat is a potent histone deacetylase inhibitor (HDACi) currently in a phase III clinical trial (www.Clinicaltrials.gov, clinical trial identifier: NCT02851797) for DMD treatment. Givinostat treatment has been effective in ameliorating morphology and muscular function in *mdx* mice. In particular, Givinostat significantly reduces fibrosis in muscle tissue and promotes the increase of cross-sectional area (CSA) of the muscles [30]. In addition, immunohistochemical analysis performed on muscle biopsies of DMD patients demonstrates that Givinostat reduces inflammation, necrosis, and fibrosis in muscle tissue and promotes increase of the CSA of the myofibers that occupy a larger fraction of muscle tissue [31]. The influence of the Ltbp4 polymorphism on the activity of Givinostat is unknown.

This study was therefore aimed at evaluating the effect of a long-term oral treatment with Givinostat (15 weeks) in the two DMD mouse models. Moreover, we also evaluated the effect of prednisone and Deflazacort in D2.B10 mice, since this strain bears a closer resemblance to human DMD pathology [15, 32]. To evaluate the efficacy of Givinostat and steroid treatment, we assessed muscle function and mice fatigability using in vivo behavioral tests (i.e., grip strength and treadmill apparatus). Histopathological analyses were also performed to assess the impact of Givinostat and steroid treatment on tissue morphology in terms of CSA, centralized nuclei, and fibrosis by histopathological analysis. We show that Givinostat has a significant and dose-dependent effect on muscle function in both models suggesting a potential benefit also on patients with the poor prognosis *LTBP4* genotype. In addition, our dose-response studies shed light on possible correlations between efficacy, histologic parameters, and pharmacokinetic (PK) analysis that could lead to further dose optimizations in the treatment of DMD patients.

## Materials and methods

### Animal experiments

#### Study approval

Procedures involving animals and their care were carried out in conformity with institutional guidelines in compliance with national and international laws and policies (Italian Governing Law: D.lgs 26/2014 “Attuazione della direttiva 2010/63/UE sulla protezione degli animali utilizzati a fini scientifici”). The research project has been authorized by the Italian Ministry of Health.

#### Animals and study design

Mice were kept under pathogen-free conditions with a 12-h light/12-h dark cycle at a temperature of 22° ± 2° and 55% ± 10% humidity. Each cage was enriched with a mouse house. Mice were regularly checked by a certified veterinarian who was responsible for health monitoring, animal welfare supervision, experimental protocols, and procedure revision. All mice were maintained under a controlled diet (VRF1 diet, Charles River), with a daily amount of chow of 4–5 g/mouse throughout the experiment [33] and received water ad libitum.

For the efficacy studies, C57BL/10 J (Stock No: 000665) wild type (wt) and C57BL/10ScSn-Dmd^*mdx*^/J (Stock No: 001801) 7–8-week-old male mice, DBA/2J wt (Stock No: 000671), and D2.B10-Dmd^*mdx*^/J (Stock No: 013141) 6–7-week-old male mice were purchased from The Jackson Laboratories (Bar Harbor, Maine, USA). After 5 days of acclimatization in the animal facility, mice were randomized into different treatment groups based on their BW. C57BL/10J wt mice were assigned to the naive wt group (healthy mice that received no treatment, but were subjected to functional tests), whereas *mdx* mice were assigned to the following groups (9 mice/group; 4–5 mice/cage): naive *mdx* (mice that received no treatment, but were subjected to functional tests), vehicle (0.5% methylcellulose, p.o.), and Givinostat (0.1, 0.3, 1, 5, 10, 25, and 37.5 mg/kg, p.o.). DBA/2J mice were assigned to the naive wt group (healthy mice that received no treatment, but were subjected to functional tests), whereas D2.B10 mice were assigned to the following groups (12–13 mice/group; 4–5 mice/gage): vehicle (filtered tap water, p.o.), Givinostat (1, 5, 10, and 37.5 mg/kg, p.o.), prednisone 1 mg/kg, and Deflazacort 1 mg/kg (i.p.).

For the PK study, C57BL/10ScSn-Dmd^*mdx*^/J (Stock No: 001801) 9-week-old male mice were purchased from The Jackson Laboratories (Bar Harbor, Maine, USA). After 5 days of acclimatization in the animal facility, *mdx* mice were randomized into 4 treatment groups (28 mice/group; 4 mice/cage) and 1 untreated group (4 mice) based on their BW. Animals were administered with single oral dose of Givinostat at 5, 10, 25, and 37.5 mg/kg and samples were collected at the following time points: 0.5, 1, 1.5, 2, 3, 4, and 6 h after administration.

#### Drug treatments

In the efficacy studies, Givinostat powder (ITF2357) was suspended in 0.5% methylcellulose (Sigma-Aldrich) and administered p.o. (by gavage) at the doses of 0.1, 0.3, 1, 5, 10, 25, and 37.5 mg/kg qdx5x15weeks (administration volume per mouse: 10 mL/kg) in *mdx* mice (see Additional Figure 1A). Vehicle-treated *mdx* mice received 0.5% methylcellulose suspension. Givinostat suspension was stored at + 4 °C, and it was freshly prepared every 7 days. Probably due to the impairment of their tongue and masticatory muscles, the oral gavage procedure turned out to be difficult to perform, and chronic treatment of D2.B10 mice by this route was considered to bear a high risk of damaging the esophagus. For this reason, Givinostat was administered in the drinking water. Givinostat was dissolved in filtered tap water and was administered in drinking water (throughout 24 h) for 105 days at the dose of 1, 5, 10, and 37.5 mg/kg/day, according to a daily estimate of water consumption of 4 ml/mouse/day [34]. Water consumption was weekly monitored throughout the entire duration of the study weighing the bottle of each cage (maximum 4 mice per cage). Prednisone and Deflazacort powder (Sigma-Aldrich) was dissolved in 2% DMSO (Sigma-Aldrich) and diluted in sterile saline on injection days. Both drugs were administered weekly (for 15 weeks) by i.p. injection at the dose of 1 mg/kg [15] in a volume of 10 mL/kg. Givinostat and steroid treatments started on days 7 and 9 of the experimental plan, respectively (see Additional Figure 1B). All mice were monitored daily and tolerability was evaluated on the basis of BW and clinical signs. No treatment-related clinical signs were observed at any time point during the studies.

In the PK study, formulations were prepared suspending Givinostat in 0.5% methylcellulose at the concentrations of 0.5, 1, 2.5, and 3.75 mg/mL. The administered volume was 10 mL/kg to obtain 5, 10, 25, and 37.5 mg/kg dosages.

### Assessment of functional tests for the evaluation of treatment efficacy

#### Maximal normalized strength

In both efficacy studies, the effectiveness of treatments was evaluated every week by measuring the forelimb strength by a grip strength meter (Ugo Basile SRL, Italy). BW of *mdx* (see Additional Figure 2) and D2.B10 mice (see Additional Figure 3) was also measured in order to normalize the absolute grip strength of each mouse with respect to its BW. The protocol provides 5 measurements for each mouse (the procedure was compliant with the standard operating procedures of TREAT–NMD Neuromuscular Network) [35]. The highest recorded value of maximal normalized strength (FNmax) obtained for each mouse at all the time points (from T0 to T15) was used for further analysis.

#### Treadmill exercise

Run to exhaustion performance of *mdx* and D2.B10 mice was evaluated every 14 or 21 days, respectively, using a treadmill apparatus (Ugo Basile SRL, Italy). The exhaustion protocol consisted in an initially horizontal running at 5 m/min for 5 min after which the speed was increased 1 m/min every minute until exhaustion (the procedure was compliant with the standard operating procedures of TREAT–NMD Neuromuscular Network) [36]. The test was concluded when mice remained for more than 10 s on the shocker plate (for both C57BL/10 J and *mdx* mice) or when D2.B10 mice reached a maximum number of shocks. Due to the excessive stress caused to D2.B10 mice by this functional test, a maximum number of 600 and 150 shocks was set. These experimental conditions were also applied to the DBA/2J wt healthy mice.

After taking baseline readings for behavioral assays (FNmax and distance run by the animals), mice were re-randomized based on these two parameters, also considering the previous randomization performed on their BW.

The grip strength and run to exhaustion tests were performed during the light-cycle phase, in the early hours of the morning (8:00–11:30 am). Both the functional tests have been conducted after a training period of 3 and 4 days, respectively, during which mice become familiar with the procedures. The training period to the functional tools started after the acclimatization period (see Additional Figure 1A and B).

### Pharmacokinetic study

#### Blood, plasma, and muscle collection

Mice were maintained under isoflurane anesthesia. Blood samples were collected in two aliquots from the retro-orbital plexus of mice and transferred into tubes containing heparin as anticoagulant (100 USPunits/mL) and then mice were sacrificed by cervical dislocation. One aliquot of 50 μL was diluted with the same volume of water and immediately frozen in dry ice. This blood was analyzed in order to subtract the blood content of Givinostat from the muscle homogenate. The other aliquot of blood was centrifuged for 10 min at 13,000 rpm at 4 °C and plasma was separated and frozen in dry ice. The quadriceps of the right hindlimb was removed, weighed, and then washed twice in 1 mL of PBS pH 7.4 and dried with paper, then it was frozen in dry ice.

#### Plasma analysis

Givinostat was determined in mouse plasma (50 μL) following a protein precipitation (200 μL of 1% HCOOH in acetonitrile) and Ostro (Waters) plate filtration. The organic phase was evaporated under a stream of nitrogen; the residue was re-dissolved in the reconstitution solvent (100 μL of 25% CH3CN-75% H_2_O-0.05% TFA), and 5 μL was analyzed. Givinostat concentrations were measured by LC-MS/MS method, in the calibration range of 0.5–400 ng/mL.

#### Blood analysis

Givinostat was determined in diluted blood (100 μL, 1:2 in water) with a liquid-liquid extraction. Samples were mixed and extracted with 2 mL of diethyl ether and centrifuged, and the organic phase was separated and evaporated under a stream of nitrogen. The residue was re-dissolved in the reconstitution solvent (150 μL of 25% CH3CN-75% H_2_O-0.05% TFA), sonicated, and filtered (regenerated cellulose syringe filters), and 5 μL was analyzed by LC-MS/MS method. The calibration range of the method was 1–200 ng/mL.

#### Quadriceps analysis

Mouse quadriceps were homogenized in PBS pH = 7.4 (1:10 w/v) with ultrasonic homogenizer (Sonoplus Mini20 - Bandelin) maintaining the samples in an ice bath. Quadricep homogenate (100 μL) was added with 200 μL of 0.5% TFA in CH3CN, vortexed, and centrifuged. Then, 100 μL of supernatant was diluted with H_2_O (1:2). Samples were filtered (regenerated cellulose syringe filters) and aliquots of 5 μL were analyzed. Givinostat concentrations were measured by LC-MS/MS method, in the calibration range of 1–40 ng/mL.

#### Calculation

PK parameters were evaluated on mean curves using conventional non-compartmental methodology by the software KineticaTM v. 5.1. Quadriceps concentrations were expressed as nanograms per gram of muscle, this value was obtained by multiplying the homogenate concentrations obtained for the homogenate volume, then subtracting the residual blood content, as reported in literature [37], considering the measured blood concentrations and dividing for the muscle weight.

### Histological analysis

#### Tissue preparation, sectioning, and staining

Tibialis anterior (TA), gastrocnemius (GAS), DIA, and heart were collected from mice maintained under isoflurane anesthesia and sacrificed by cervical dislocation. These muscles were collected from C57BL/10J wt and *mdx* (5 mice/group) at the end of treatments and from DBA/2J wt and D2.B10 mice (5 mice/group) at two different time points to be examined by a pathologist (for more details see Additional Table 1). Due to the results obtained by the functional tests, muscles from only some treatment groups were analyzed in both DMD models (see Additional Table 1). All the collected muscles were fixed in 10% buffered formalin solution (Bio-optica, Milan, Italy) at + 4 °C for at least 48 h. After fixation, muscular samples were transversely trimmed, caged, and paraffin embedded overnight with an Automated Vacuum Tissue Processor Floor (ETP, Histo-Line Laboratories) and included in paraffin blocks. Serial transverse cross-sections (4 μm thick) were cut with a microtome (Leica RM 2255), collected onto uncoated glass slides, and stained using a standard protocol for hematoxylin and eosin (Mayer hematoxylin & Aqueous G Eosin 1%, Bio-Optica) and Sirius Red staining (Direct Red 80, Sigma-Aldrich). For the image analysis and quantitation, hematoxylin and eosin- and Sirius Red-stained slides were examined with an Olympus BX51 light microscope. From each sample, 1 to 3 random microphotographs at the magnification of × 4 and × 10 (TA, GAS and heart) or × 20 (DIA) were collected using Image-Pro Plus system. The digital images were processed by a pathologist using ImageJ software (U.S. National Institutes of Health, Bethesda, MD, USA). To quantify CSA and centralized nuclei (%) on hematoxylin and eosin-stained sections, one hundred myofibers were manually measured for each muscle/mouse using a tablet pen (Wacom Intruos) and analyzed by the ImageJ software after a linear calibration of 150 μm. The fibrotic area, corresponding to the area stained in red, was compared to the total area of the tissue section, and the results were expressed as percentage fibrosis. The % of fibrosis of muscles in the different treatment groups was expressed by averaging the three values obtained from each muscle.

In these experiments, the histopathological evaluation of muscle inflammatory infiltrate, adipose tissue deposition, regeneration, and degeneration of the muscular tissue was also performed. The severity of myodystrophy in the different muscles (TA, GAS and DIA) was quantified through a weighted histopathological method that considered some parameters scored by severity and extension of the injury: muscle degeneration/necrosis, regeneration, inflammatory infiltrate, interstitial reaction, and adipose tissue deposition. Each parameter was classified by severity (mild = 1, moderate = 2, and severe = 3) and extension (focal = 1, multifocal = 2, and diffuse = 3). The individual severity score was calculated for each animal and an average score per group was calculated.

### Whole-genome miRNA expression profiling

#### Plasma collection

Blood samples were taken from the retro-orbital plexus of mice maintained under isoflurane anesthesia. Blood was placed in tubes containing 50 μL (for 1 mL of total blood) of EDTA (100 nM) and then it was centrifuged at 13,300 rpm at + 4 °C for 10 min. Plasma was separated (400–500 μL) and immediately frozen in dry ice and stored at − 80 °C until analysis.

These analyses were carried out by Biogazelle (Technologiepark 82, 9052 Zwijnaarde, Belgium).

Thirteen mouse plasma samples (naive wt, n = 3; naive *mdx*, n = 3; vehicle, n = 4; Givinostat 37.5 mg/kg, n = 3) were delivered to Biogazelle in dry ice.

#### RNA extraction

RNA was isolated using the miRNeasy Serum/Plasma Kit (Qiagen) following the manufacturer’s instructions.

#### Small RNA sequencing and data processing

Libraries for small RNA sequencing were prepared using the NEBNext small RNA library prep kit (New England Biolabs) according to the manufacturer’s instructions. Briefly, 5 μl of RNA eluate was used as input for RNA adapter ligation (using 3′ and 5′ RNA adapters) followed by reverse transcription and PCR amplification with bar-coded primers. Size selection of individual libraries was performed on a Pippin Prep system (Sage Science) to recover the ~ 147–157-nt fractions containing mature miRNAs. Next, qPCR-based normalization was used for pooling of the individual small RNA libraries. Finally, the resulting small RNA pools were concentrated via ethanol precipitation and quantified using the Qubit 2.0 fluorometer (Thermo Fisher Scientific) prior to sequencing with read length of 75 bp on a NextSeq 500 sequencer (Illumina).

Sequencing reads were filtered based on stringent read quality control. After adapter trimming with Trimmomatic, reads were collapsed and mapped to the reference genome (GRCm38) using Bowtie [38]. Reads up to 25 nucleotides are mapped with no mismatches, while longer reads (mainly from RNA species other than miRNAs) are mapped allowing a maximum of 2 mismatches. Using genome annotation data from Ensembl (release 84), UCSC (mm10), and miRbase (release 21), mapped reads were subsequently annotated to mature miRNAs and other small RNA species including tRNA fragments, rRNA, sn(o)RNAs, and piRNAs. Raw count data is reported for each isomiR and each mature miRNA (as the sum of all isomiR reads from the canonical mature miRNA locus). Raw miRNA reads were normalized using the geometric mean-based method implemented in DESeq2. Spectral maps were generated using mpm R package [39] based on DESeq2 normalized counts [40]. In this study, the contrasts analyzed were the following: naive *mdx* vs naive wt (T16) and Givinostat 37.5 mg/kg vs vehicle (T16).

### Statistical analysis

Statistical analysis was performed using a GraphPad Prism version 8 software.

All experimental data were expressed as mean ± standard error (s.e.). Multiple statistical comparisons between groups were performed by two-way ANOVA (mice BW, FNmax, and distance run) and by one-way ANOVA (centralized nuclei and fibrosis percentage), with Bonferroni’s multi-comparison test. P values ≤ 0.05 were considered as statistically significant and are indicated in each figure legend; p values > 0.05 were considered as statistically not significant (ns).

#### CSA statistical analysis

The deciles of the CSA of the animals treated with Givinostat at the different doses were plotted against the corresponding deciles of the vehicle-treated mice and a linear regression analysis was performed on natural [CSAtreated = b CSAcontrol + a] and log_transformed data [log(CSAtreated) = log(a) + b log(CSAcontrol)]. If the shape of the two distributions is not significantly altered by Givinostat treatment, the value of the slope (b) should not differ significantly from 1; in addition, if the value of the intercept (a) is significantly different from zero, the presence of a shift (S) between the two CSAtreated and CSAcontrol distributions may be considered. In case of untransformed data, this means that the effect of Givinostat treatment is additive and any decile of the CSAtreatment distribution may be derived from the control data summing up a constant quantity (S) to each fiber AREA of the control group (CSAtreated = CSAcontrol + S). In case of log_transformed data, the effect of Givinostat has to be considered as a multiplicative factor on any generic fiber of the muscle under evaluation, whose size, after the treatment, results K times larger in comparison to the control group value (CSAtreated the S or K values = K × CSAcontrol, with K = 10^a). The S or K parameter values were then determined through an iterative estimation procedure, based on sum of squares (SSQ) minimization, directly comparing the CSA distribution observed in treated animals with that obtained summing up the S value or multiplying by K the CSA values of the control group.

Differential miRNA abundance was performed using the same DESeq2 package. A false discovery rate (FDR) of 5% was applied on p values adjusted according to the Benjamini and Hochberg method.

DESeq2 applies a method for outlier detection [41]. These miRNAs have reads in at least one sample but are ignored for the statistical test (hence no p value). Furthermore, DESeq2 applies a filtering on low counts; these miRNAs have reads in at least one sample but have a low average abundance. The statistical test is applied for these miRNAs—they receive a p value, but they are excluded from multiple test correction—they receive no adjusted p value.

## Results

### Givinostat improves muscle function in a dose-dependent way in mdx mice

In order to start dissecting the influence of Ltbp4 polymorphism on the efficacy of Givinostat in DMD mouse models, we first set out to characterize the effect of our HDAC inhibitor in *mdx* mice. Muscle function was investigated using both the grip test and the treadmill exhaustion test.

#### Grip test

**Fig. 1:**
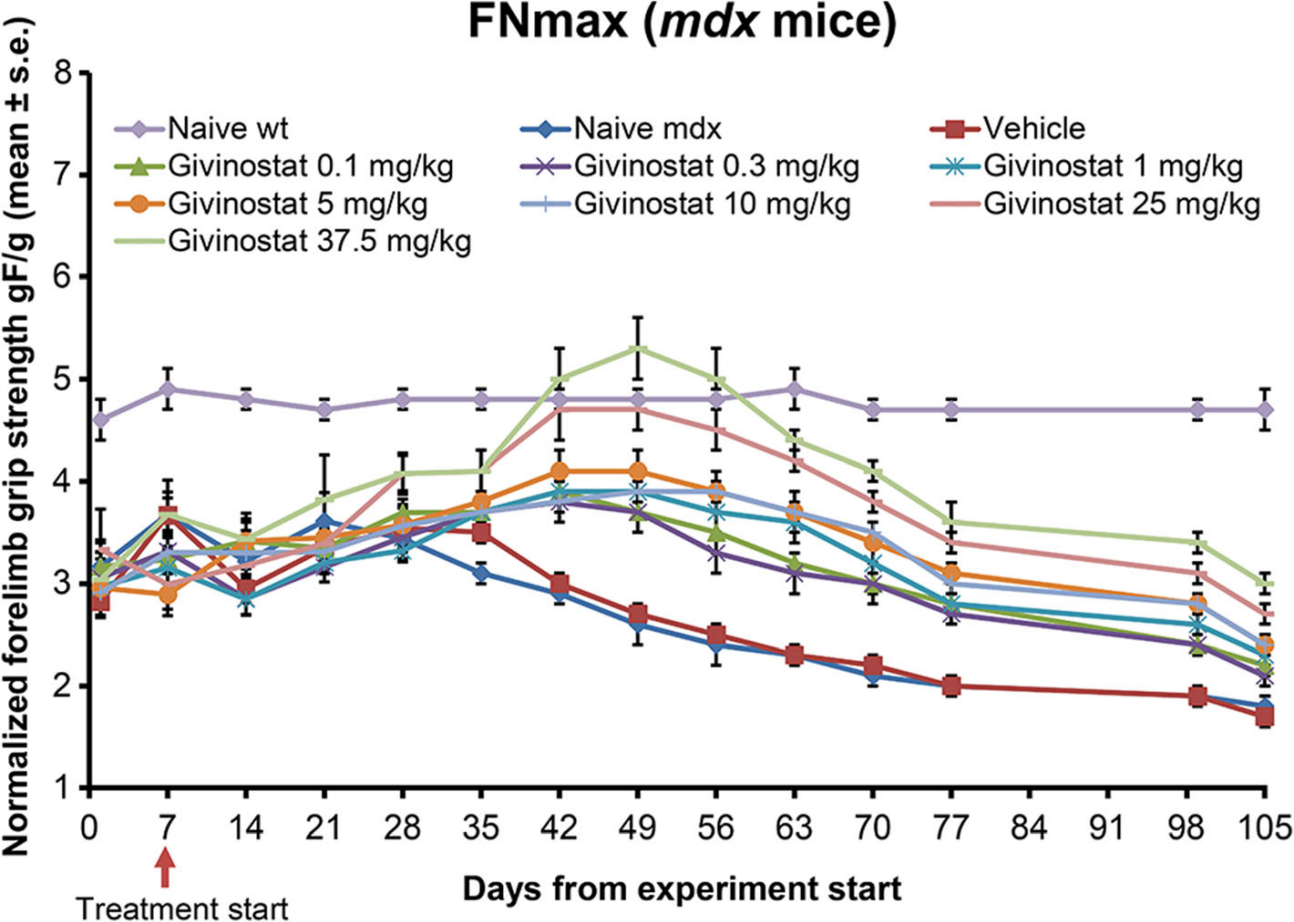
Givinostat increases maximal normalized strength (FNmax) in *mdx* mice in a dose-response manner. Givinostat treatment started on day 7. Data are expressed as the mean ± standard error (n = 9; wt: wild type)

Untreated *mdx* mice showed a small increase in maximal normalized strength up to day 21 from the baseline value (day 0), followed by a progressive decline (Fig. 1). *Mdx* vehicle-treated mice behaved as the *mdx* naive mice, clearly indicating that the vehicle did not exert any effect. In this setting, Givinostat treatment induced a remarkable, dose-dependent increase of the FNmax with the maximal efficacy observed at the dose of 37.5 mg/kg. On treatment day 49, this dose transiently improved FNmax to a level that was comparable to that of wt healthy mice (Fig. 1). From this day on, the animals started to diminish the developed strength but the beneficial effect of Givinostat remained significant up to day 105 (see Additional Table 2).

#### Treadmill exhaustion test

**Fig. 2:**
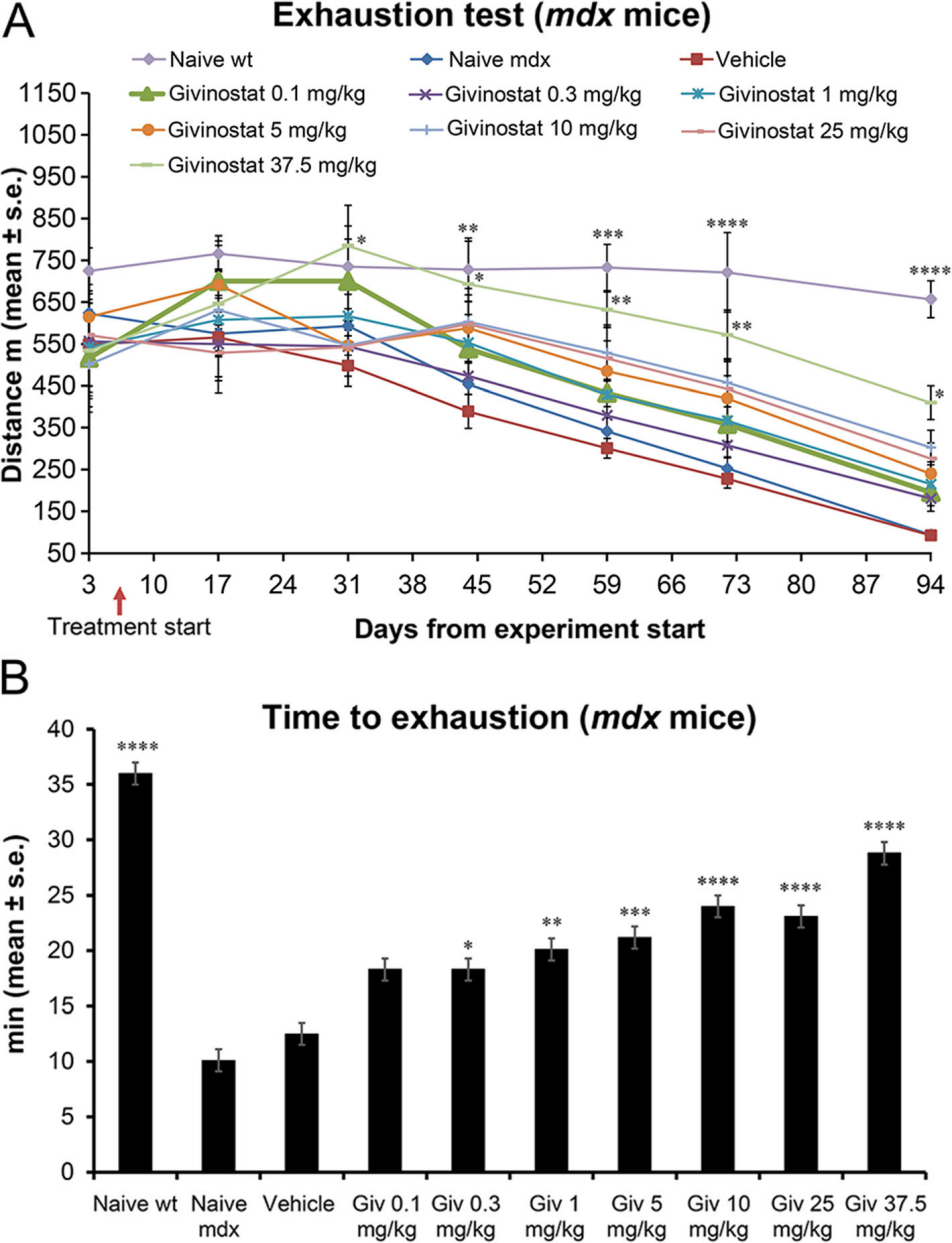
Givinostat increases distance run by mice (**A**) and time to exhaustion at day 94 (**B**) in *mdx* mice. Givinostat (Giv) treatment started on day 7. Data are expressed as the mean ± standard error (2-way ANOVA with Bonferroni’s multiple comparison test (A) and one-way ANOVA with Bonferroni’s multiple comparison test (B), *p < 0.05; **p < 0.01; ***p < 0.001; ****p < 0.0001 vs Vehicle; n = 9; wt: wild type)

Givinostat treatment at the dose of 37.5 mg/kg significantly enhanced the performance of mice on day 31 and, between days 31 and 44, their performance (785 ± 97 and 693 ± 103 m, respectively) was superimposable on that of wt mice (735 ± 66 and 728 ± 76 m on days 31 and 44, respectively). On day 94, mice were able to run for 410 ± 40 m (the distance covered by wt mice was 657 ± 44 m), whereas the *mdx* vehicle mice ran for only 92 ± 4 m (Fig. 2A). Lower doses did not reach significance, although a trend of a dose-response effect was evident throughout the entire experimental period (Fig. 2A). In addition to the distance, we also analyzed the time to exhaustion at the last time point (day 94). Givinostat, at the highest dose, significantly prolonged the time to exhaustion (Fig. 2B). The effect was also significant at lower doses, with 0.3 mg/kg being the lowest effective dose.

### Pharmacokinetics of Givinostat in C57BL/10 mdx mice

**Fig. 3:**
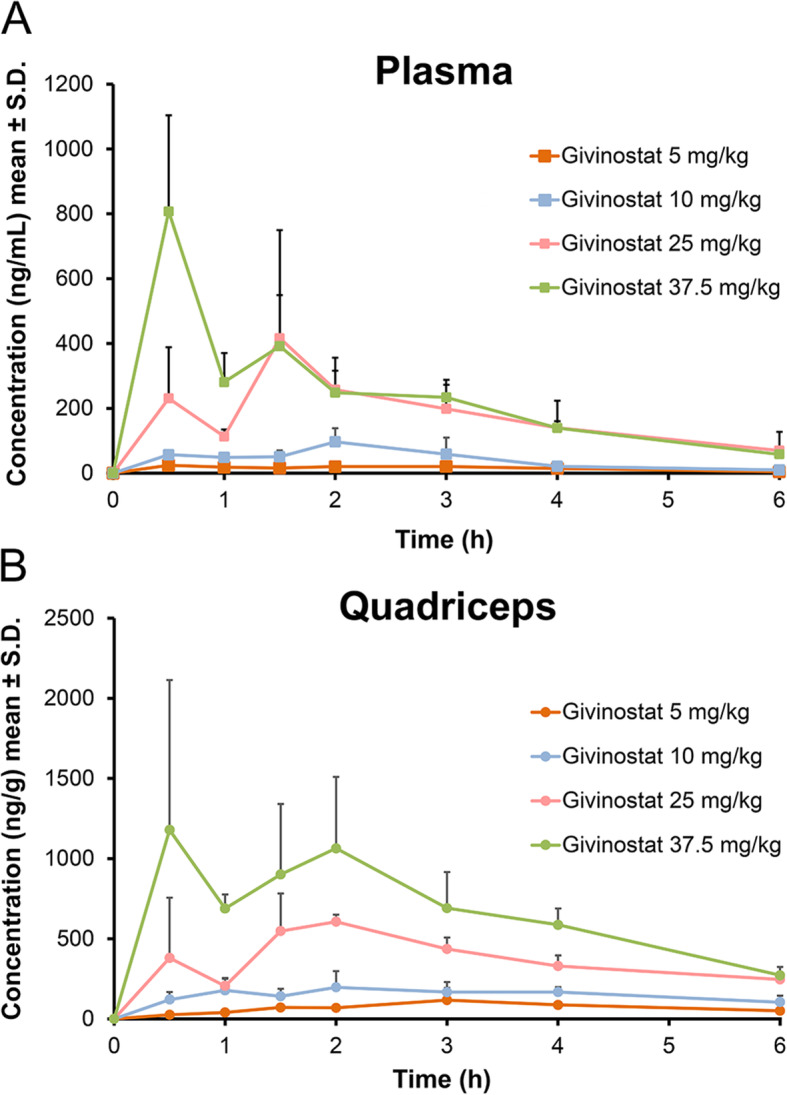
Plasma and quadriceps Givinostat concentrations in *mdx* mice. Linear plot of plasma (**A**) and quadriceps (**B**) levels of different doses of Givinostat orally administrated to *mdx* mice (n = 4)

The functional data reported thus far, point to a dose-dependent improvement of functional tests by Givinostat. We next investigated the correlation with systemic and tissue-specific drug exposure by measuring blood, plasma, and muscle Givinostat levels after single oral administration of different doses of the drug. Mean concentration levels of Givinostat in plasma and quadriceps are shown in Fig. 3 (Fig. 3A,B, respectively), and mean PK parameters in plasma, blood, and quadriceps are reported in Table 1 (Table 1). Givinostat was detectable in all plasma, blood, and muscle samples up to 6 h after the oral treatment at all doses investigated.

**Table 1:**
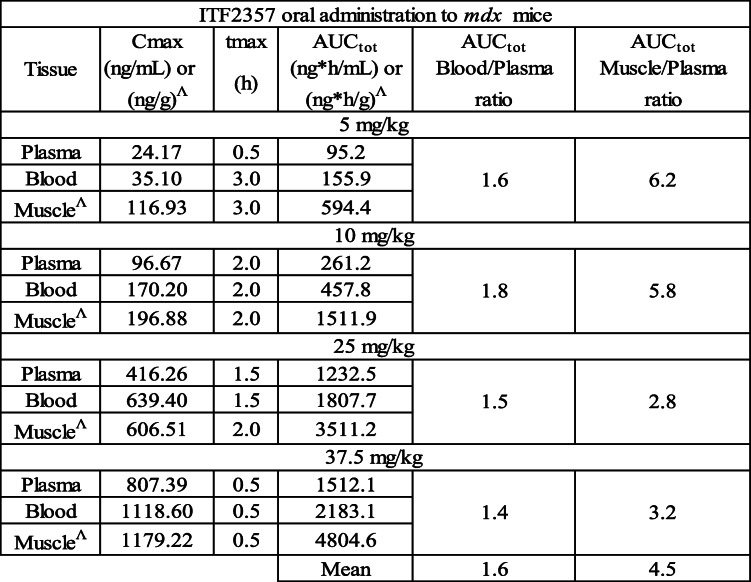
Quantification of pharmacokinetic parameters

^Mean PK parameters for Givinostat in plasma, blood and muscle after oral administration of different doses to *mdx* mice

Blood quantification was mainly performed in order to subtract from muscle homogenate the amount of Givinostat in the residual blood contained in the muscle, according to the physiological parameters reported in literature [37]. The level of Givinostat in blood was always higher than in plasma indicating a distribution of the drug into blood cells. The AUC ratio was calculated and a mean value of 1.6 was found. Similar PK profiles both in plasma and in quadriceps were also found without a delay time for tissue distribution. The AUCs of Givinostat in the muscle were always higher than in plasma, indicating a preferential distribution of Givinostat into muscle tissue.

The results of the PK study show that, upon oral administration, Givinostat exposures increase in a dose-dependent way both in terms of Cmax and AUC and that the compound quickly distributes into muscular tissue with an AUC about 4.5 times higher than in plasma. The efficacy of Givinostat may well be related to these high exposure levels in the muscular tissue.

### Histopathological analysis

**Fig. 4:**
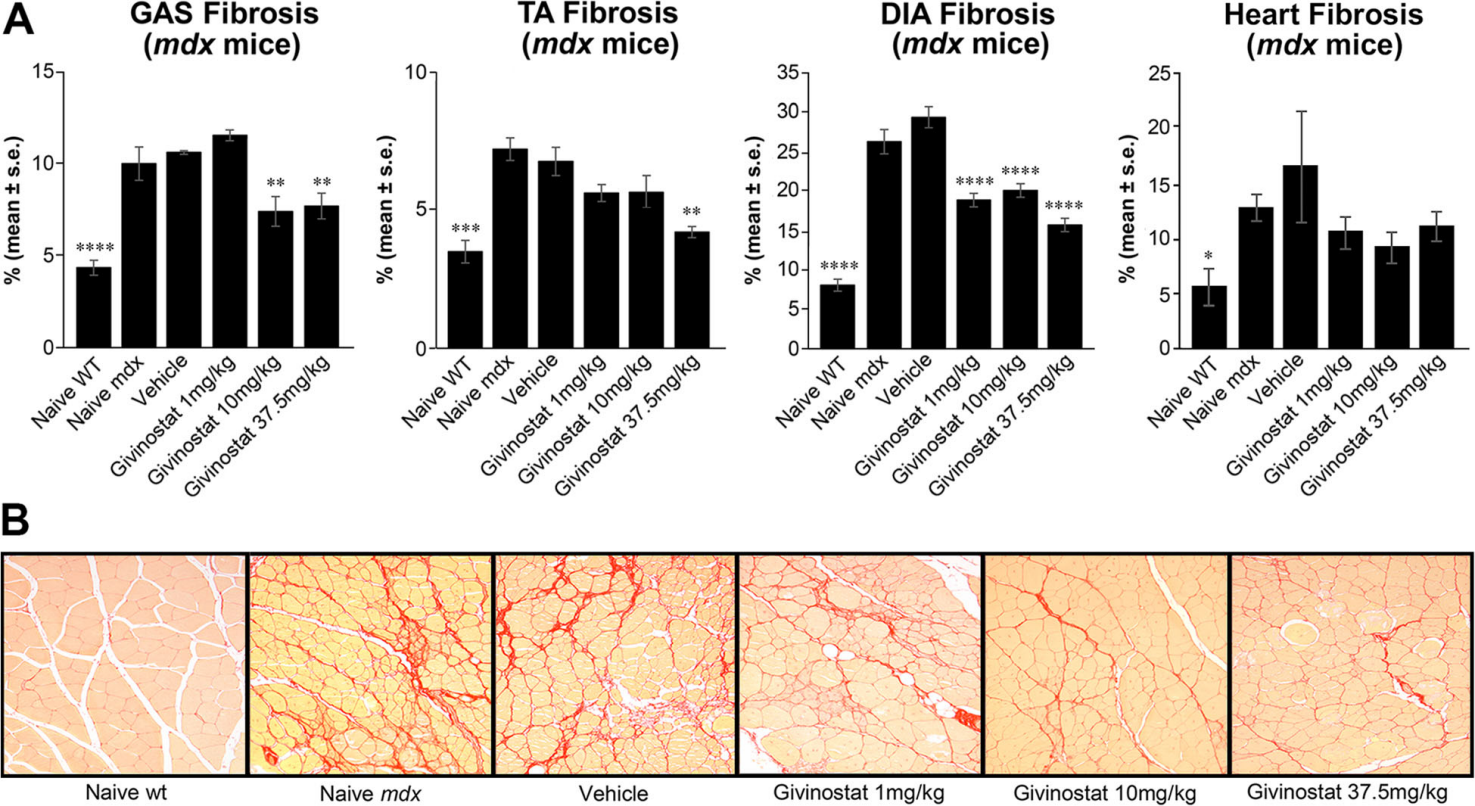
Effect of Givinostat treatment on muscle fibrosis in *mdx* mice. Givinostat significantly reduces fibrosis in gastrocnemius (GAS), tibialis anterior (TA), and diaphragm (DIA) muscles of *mdx* mice. In the heart, the effect of Givinostat was not significant. Analysis was performed at T16 time point. Data are expressed as the mean ± standard error (1-way ANOVA with Bonferroni’s multiple comparison test, *p < 0.05; **p < 0.01; ***p < 0.001; ****p < 0.0001 vs vehicle; n = 5; wt: wild type) (**A**). Representative images of Sirius Red staining of GAS muscle transverse sections of naive wild type (wt), naive *mdx*, vehicle, Givinostat 1, 10, and 37.5 mg/kg treated mice (**B**)

Histopathology was performed to investigate whether the observed functional improvements in Givinostat-treated *mdx* mice correlate with better preservation of muscle integrity at the histological level. The effect of Givinostat on fibrosis was analyzed. At the end of treatment (T16), GAS, TA, DIA, and heart in naive *mdx* mice had a significantly higher fibrosis area compared to healthy muscles. Givinostat administered at 10 and 37.5 mg/kg significantly reduced fibrosis (by about 30%) in GAS, whereas an effect on fibrosis in TA was observed only at the dose of 37.5 mg/kg (Fig. 4A). In the DIA, all the 3 analyzed doses of Givinostat significantly reduced the amount of fibrosis by about 40% (Fig. 4A). In the heart, the amount of fibrosis seems to be more variable than in other muscles. However, vehicle-treated *mdx* mice seem to have a significantly higher amount of fibrosis compared to that of naive wt mice. Givinostat reduced fibrosis at all the 3 doses analyzed compared to that of vehicle-treated mice, but the effect was not significant (Fig. 4A). Figure. 4B shows an example of the reduction of fibrosis (visible through the reduction of red as the dose of Givinostat increases) in histological sections of GAS stained by Sirius Red staining.

**Fig. 5:**
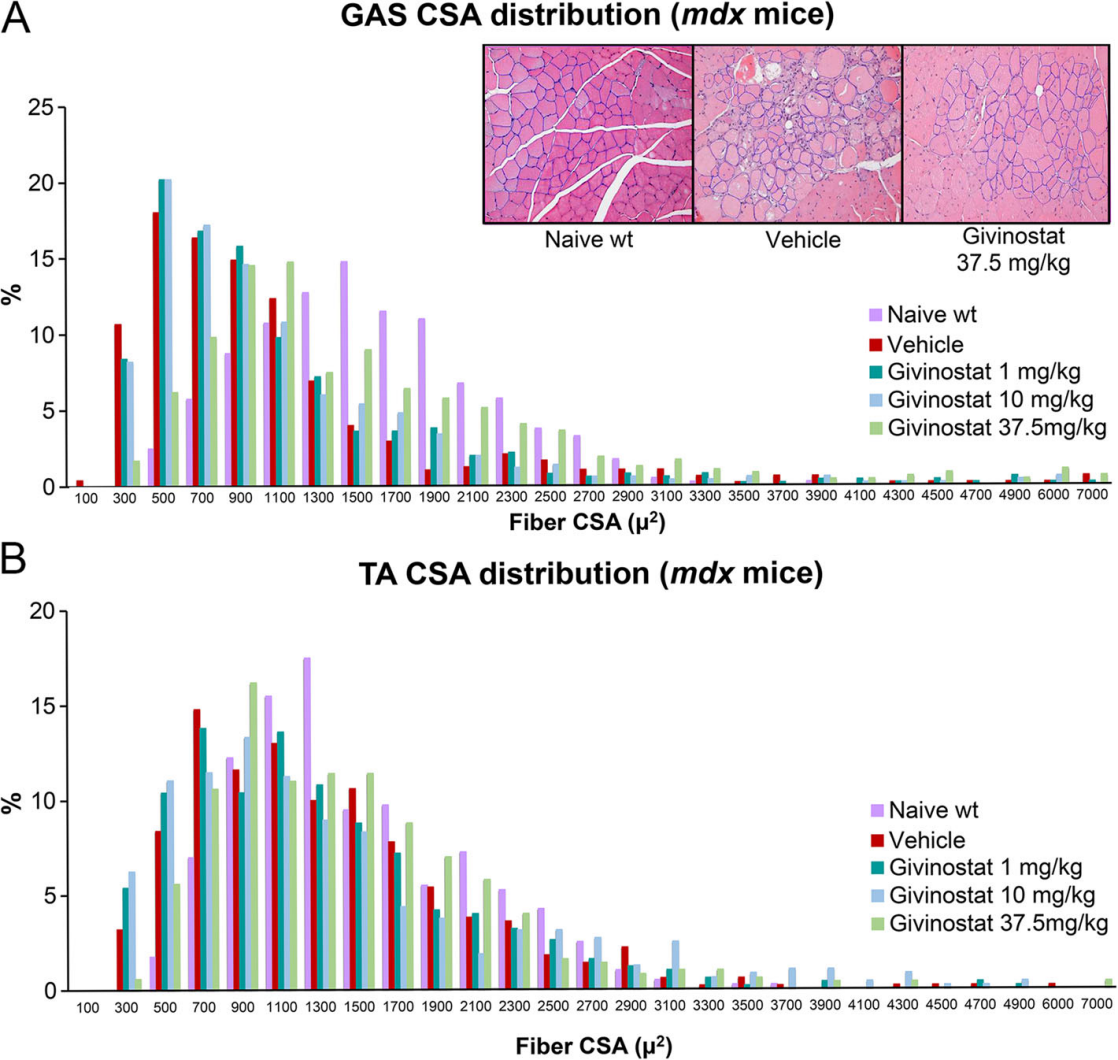
Graph of the analysis of myofiber cross-sectional area (CSA) of muscles in *mdx* mice. Frequency graphs showing fiber CSA distributions in gastrocnemius (GAS) (**A**) and tibialis anterior (TA) (**B**) (5 mice/group, except for Naive wild type (wt) and Naive *mdx* groups, 4 mice/group). Each distribution was calculated as percentage of the total number of cases to account for the different number of cases within the groups. In Fig. 5A are also reported three examples of hematoxylin and eosin-stained transverse sections of myofibers areas in GAS of Naive WT, vehicle, and Givinostat 37.5 mg/kg treated mice

The effect of Givinostat treatment on myofiber cross-sectional areas was also evaluated. Determination of fiber CSA of GAS in *mdx* mice revealed an increase in fiber size after 105 days of Givinostat treatment at 37.5 mg/kg, when compared to vehicle-treated mice. GAS CSA of Givinostat-treated mice have doubled GAS CSA values (peak at 1100 μm^2^) as compared to those of *mdx* vehicle GAS (peak at 500 μm^2^) (Fig. 5A). According to the statistical analysis, this difference could be explained by assuming a multiplicative model effect with a 1.72 K, which provides the multiplicative factor to be applied to each fiber CSA value of the vehicle group to obtain the expected CSA distribution after Givinostat at 37.5 mg/kg treatment. Interestingly, this K value is well in line with that observed in a previously reported clinical study [31].

In TA, fiber CSA showed a limited increase after Givinostat at 37.5 mg/kg treatment compared to vehicle-treated mice, with a shift toward wt CSA values (1400 μm^2^). TA CSA of Givinostat at 10 and 37.5 mg/kg treated mice have a peak value of 900 μm^2^ similar to that of *mdx* vehicle TA (700 μm^2^) (Fig. 5B). Statistical analysis of the TA CSA distribution confirmed the results observed using the additive model effect, with the shift effect parameter S = 140 μm^2^.

In DIA, no major change in CSA distribution was evident in mice treated with Givinostat 1, 10, and 37.5 mg/kg compared to vehicle-treated mice (CSA peak value in vehicle-treated mice was 550 μm^2^, the same observed in Givinostat 37.5 mg/kg treated mice).

The percentage of centralized nuclei on muscle tissue was also analyzed. Centralized nuclei are considered a normal pattern of myofiber regeneration, even though this parameter is difficult to interpret because centralized nuclei may persist even after muscle repair. Nuclear centralization was absent in GAS, TA, and DIA muscles from 24 to 25-week-old wt mice, while it was very diffuse in GAS (77 %), TA (66 %), and DIA (58 %) of vehicle-treated *mdx* mice at the same age. No marked treatment-related effects were observed: Givinostat at 1, 10, and 37.5 mg/kg treated mice showed a similar nuclear centralization to that of vehicle-treated mice (67.6 ± 2.4%, 58 ± 3%, and 61.3 ± 3.8%, respectively, in GAS; 71 ± 1.6%, 73.2 ± 3.7%, and 60.8 ± 2.5%, respectively, in TA; 57 ± 2.9%, 61.4 ± 3.5%, and 41.4 ± 4.5%, respectively, in DIA).

As reported in the literature, the high percentage in centralized nuclei in *mdx* mice is due to their characteristic mechanism of continuous regeneration after muscle degeneration [26] and could also explained a lack of marked effect in treated mice.

The histological evaluation of muscle inflammatory infiltrate, adipose tissue deposition, regeneration, and degeneration of the muscular tissue was not significantly different among the treatment groups. In particular, the degree of inflammatory infiltrate and adipose tissue deposition was very limited in all samples and, therefore, not useful for discriminating the efficacy of the different doses of Givinostat (Additional Table 3).

Functional and histological improvements as a function of administered doses are summarized in Additional Table 4 (see Additional Table 4).

### Whole-genome miRNA expression profiling

MicroRNAs (miRNAs) are small, approximately 22 nt, non-coding RNA molecules that post-translationally regulate gene expression. Several miRNAs were described to be involved in skeletal muscle proliferation, differentiation, and regeneration [42]. In addition, miRNAs were proposed to be useful serum biomarkers for muscular dystrophy [43]. Previous publications have implicated miRNAs as mediators of exosome-regulated biological processes [44–46]. Exosomes, or extracellular vesicles, are gaining a lot of attention in the scientific community due to their role in cell-cell communication. Fibro-adipogenic progenitor (FAP)-derived exosomes accumulate in the interstitial space of regenerating muscles and can mediate miRNA transfer to MuSCs. In vivo exposure to HDAC inhibitors was reported to lead to an enrichment of specific miRNAs in FAP-derived exosomes and increased levels of exosomal miR-206 were required to promote MuSC activation and expansion ex vivo and to stimulate regeneration of dystrophic muscles in vivo. These data point to the importance of investigating miRNA levels in response to Givinostat treatment in vivo. To investigate whether the observed effects of Givinostat on muscle function and histology correlate with changes in plasma miRNA levels, we used the RNAseq approach to evaluate the whole-genome miRNA expression profile in plasma of *mdx* mice treated with Givinostat at the dose of 37.5 mg/kg. In this study we evaluated the differential expression of miRNA between selected groups of samples collected at T16. The number of replicates per group were compared in a pairwise manner. The compared conditions (i.e., contrasts) and the number of up- and downregulated miRNAs are listed in Additional Table 5 (see Additional Table 5).

We focused on the most highly modulated miRNAs with known association with dystrophic disease at T16 for the naive *mdx* vs naive wt contrast. The analysis of these two groups is instructive to comprehend if the epigenetic treatment ameliorates the *mdx* phenotype by restoring the altered expression of certain miRNAs. To evaluate the effect of Givinostat on the modulation of miRNAs, we chose the last treatment (T16) and the most efficacious dose (37.5 mg/kg) vs vehicle contrast.

**Fig. 6:**
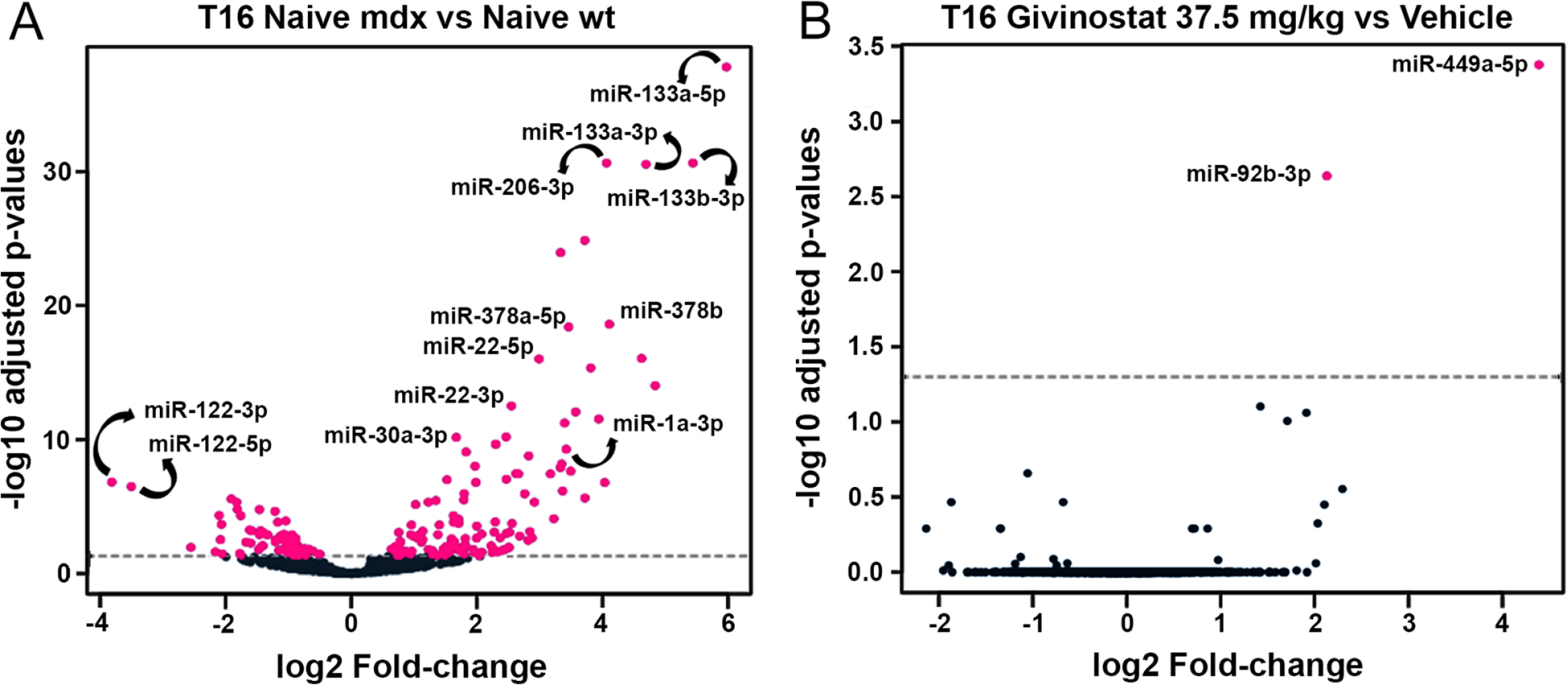
Volcano plots of differentially expressed miRNA in wt and *mdx* mice. The analysis was performed for Naive *mdx* vs Naive wild type (wt) (**A**) and Givinostat 37.5 mg/kg vs vehicle (**B**) contrasts after 15 weeks of treatment (T16). Log2 fold-changes estimated by DESeq2 versus –log10 adjusted p values (FDR: false discovery rate) are indicated in the plot. Black dots indicate miRNA with non-significant fold change (FDR > 0.05). Pink dots indicate significantly differentially expressed miRNAs at FDR < 0.05

In the T16 naive *mdx* vs naive wt contrast, 120 miRNAs were significantly upregulated and 66 miRNAs were significantly downregulated in plasma of *mdx* mice. Among the upregulated miRNAs we found miR-133a-5p (FC = 5.973; the most upregulated miRNA), miR-133b-3p (FC = 5.441), miR-133a-3p (FC = 4.693), miR-378b (FC = 4.109), miR-206-3p (FC = 4.065), miR-1a-3p (FC = 3.423), miR-22-5p (FC = 2.991), and miR-22-3p (FC = 2.550) (Fig. 6). All of these miRNAs have been reported to be elevated in the dystrophic mice [47, 48]. Moreover, miR-133, miR-1 and miR-206 levels were found to be elevated also in the plasma of DMD patients [49].

In mice, miR-133a-3p, miR-1a-3p, and miR-206-3p are specifically muscle-enriched miRNAs that regulate both the proliferation of myofibers and the myogenic differentiation during muscle growth [50, 51]. In particular, miR-206-3p plays a key role in dystrophic muscle regeneration and is known to be enriched in regenerating myofibers [52, 53]. The abundance of these three miRNAs correlates with the progression of dystrophic pathology [54].

The most significantly downregulated miRNAs in T16 naive *mdx* vs naive wt contrast were miR-122-3p and miR-122-5p that are probably involved in the regulation of muscle growth and lipid deposition [55], but, to our knowledge, these miRNAs were so far not described to be dystrophy-related.

Among the Givinostat-induced miRNAs (T16 Givinostat 37.5 mg/kg vs vehicle contrast), the only two that were significantly upregulated were miR-449a-5p (FC = 4.388) and miR-92b-3p. The upregulation of these two miRNAs in *mdx* muscle after HDACi treatment (trichostatin A—TSA) was already reported in the literature [56, 57].

miR-449a-5p is involved in different pathways, such as TGF-β, TNF, MAPK, Wnt, FoxO, PI3K-Akt, and Hippo signalling pathway and was proposed to be linked to stem cell pluripotency [57].

miR-92b-3p, along with miR-133a-3p, is one of the cardiac specific miRNAs involved in heart failure (i.e., cardiomyocyte apoptosis, hypertrophy, and inflammation) and was reduced in the coronary sinus of patients with heart failure [58]. In particular, miR-92b-3p was observed to be decreased in mouse hypertrophic cardiomyocytes upon angiotensin II antagonist treatment and its overexpression can attenuate the hypertrophic phenotype by downregulating the hypertrophy related genes [58–60]. *Mdx* mice show a mild cardiomyopathy and, according to the literature, we found that at T16, miR-92b-3p in naive *mdx* plasma was downregulated compared to naive wt mice (FC = − 0.896, not significant). After chronic Givinostat treatment (T16 Givinostat 37.5 mg/kg vs vehicle), our analysis showed a significant upregulation (FC = 2.130) of miR-92b-3p, suggesting a possible beneficial effect on cardiac function of *mdx* mice upon treatment with Givinostat. Since we focused exclusively on plasma miRNA levels, it will be interesting to investigate the effects of Givinostat on miRNA levels in skeletal or cardiac muscle in future studies.

The results of each contrast are displayed as volcano plots including all miRNAs in Fig. 6. Our results are in agreement with literature data that indicate a profound dysregulation of miRNA levels in skeletal muscle from *mdx* mice [57]. Givinostat treatment does not revert the overall pattern toward normalcy but specifically acts on two miRNAs that have been reported to be functionally linked to muscle physiology and more specifically to cardiac pathologies.

### Treatment efficacy evaluation in D2.B10 mouse model

Having characterized the functional, histological, and molecular effects of Givinostat on *mdx* mice with a mild dystrophic phenotype, we next evaluated the drug in the more severe D2.B10 model. We also included GC steroid treatment groups in this experiment to evaluate the efficacy of Givinostat in comparison to the clinically relevant standard of care treatment. Both prednisone and Deflazacort were used for this purpose.

Unfortunately, we observed that repeated, chronic oral administration of Givinostat by gavage turned out to be difficult and unsafe for these mice. This difficulty may be related to a more pronounced oropharyngeal or esophageal dysfunction in D2.B10 mice as compared to *mdx* mice. We noticed that feeding difficulties and oropharyngeal or esophageal dysfunctions were also reported to occur in Duchenne patients [61]. As a consequence, the drug was given in drinking water (see “Drug treatments” paragraph in “Materials and methods” section). To estimate the daily dose administered to the animals using this procedure, water consumption was measured every week during the study. We calculated the actual doses of Givinostat ingested by mice, to be in the following ranges: 0.8–1.1 mg/kg, 3.6–6 mg/kg, 7.2–11.6 mg/kg, and 27.5–41.5 mg/kg. We noticed that these data were in line with the expected doses of 1, 5, 10, and 37.5 mg/kg, respectively. An increase in the water intake by the animals, and therefore in the dose of Givinostat is probably due to the growth, and therefore, the weight gain of the animals over time.

### Functional activity measurement

In an analogy to the characterization done on the *mdx* mice, muscle function of D2.B10 mice was investigated using both the grip test and the treadmill exhaustion test.

#### Grip test

**Fig. 7:**
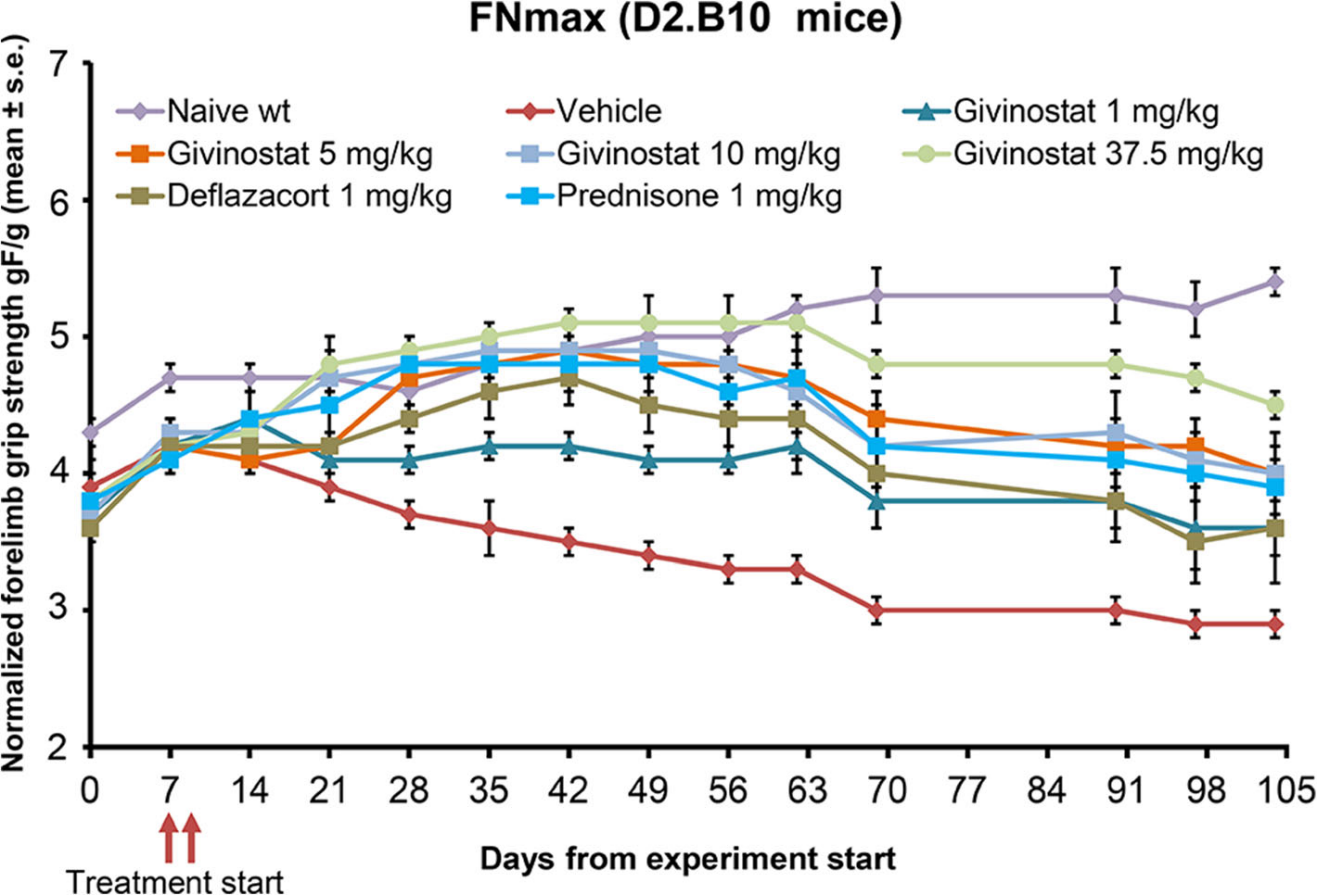
Effect of Givinostat and steroids on maximal normalized strength (FNmax) in D2.B10 mice. Givinostat and steroid treatment started on days 7 and 9, respectively (wt: wild type)

The developed FNmax in DBA/2J wt mice increased throughout the entire experimental period (Fig. 7), while FNmax in D2.B10 vehicle-treated mice tended to decrease starting from day 14 (Fig. 7), indicating a progressive impairment of muscle function. The effect of Givinostat on FNmax was dose-dependent, with maximal efficacy observed at the dose of 37.5 mg/kg. Givinostat at 10 and 37.5 mg/kg as well as prednisone treatment increased the FNmax of D2.B10-treated mice to that of wt healthy mice starting from day 21 until days 49–62 (Fig. 7). From day 62 onwards, D2.B10-treated mice started to diminish the FNmax, but the effect of Givinostat (administered at 5, 10 and 37.5 mg/kg) and prednisone remained significantly elevated until day 104 (see Additional Table 6). The lowest dose that exerted a significant effect from day 21 to day 104 on FNmax was 10 mg/kg. Deflazacort and Givinostat at 1 mg/kg significantly improved the FNmax for a limited time interval, i.e., from day 28 to day 90 and from day 42 to days 90/104, respectively (see Additional Table 6). The effect of prednisone on FNmax was comparable to that of Givinostat 5 e 10 mg/kg for all the treatment period, while the effect of Deflazacort was lower and not significantly different from prednisone. Significant differences in FNmax values between Givinostat at 37.5 mg/kg versus prednisone were observed only at days 90 and 97 (p < 0.05) and, at the same experimental time points, a significant difference was also seen between Givinostat at 37.5 mg/kg vs Deflazacort (p < 0.05 and p < 0.01, respectively).

#### Treadmill exhaustion test

**Fig. 8:**
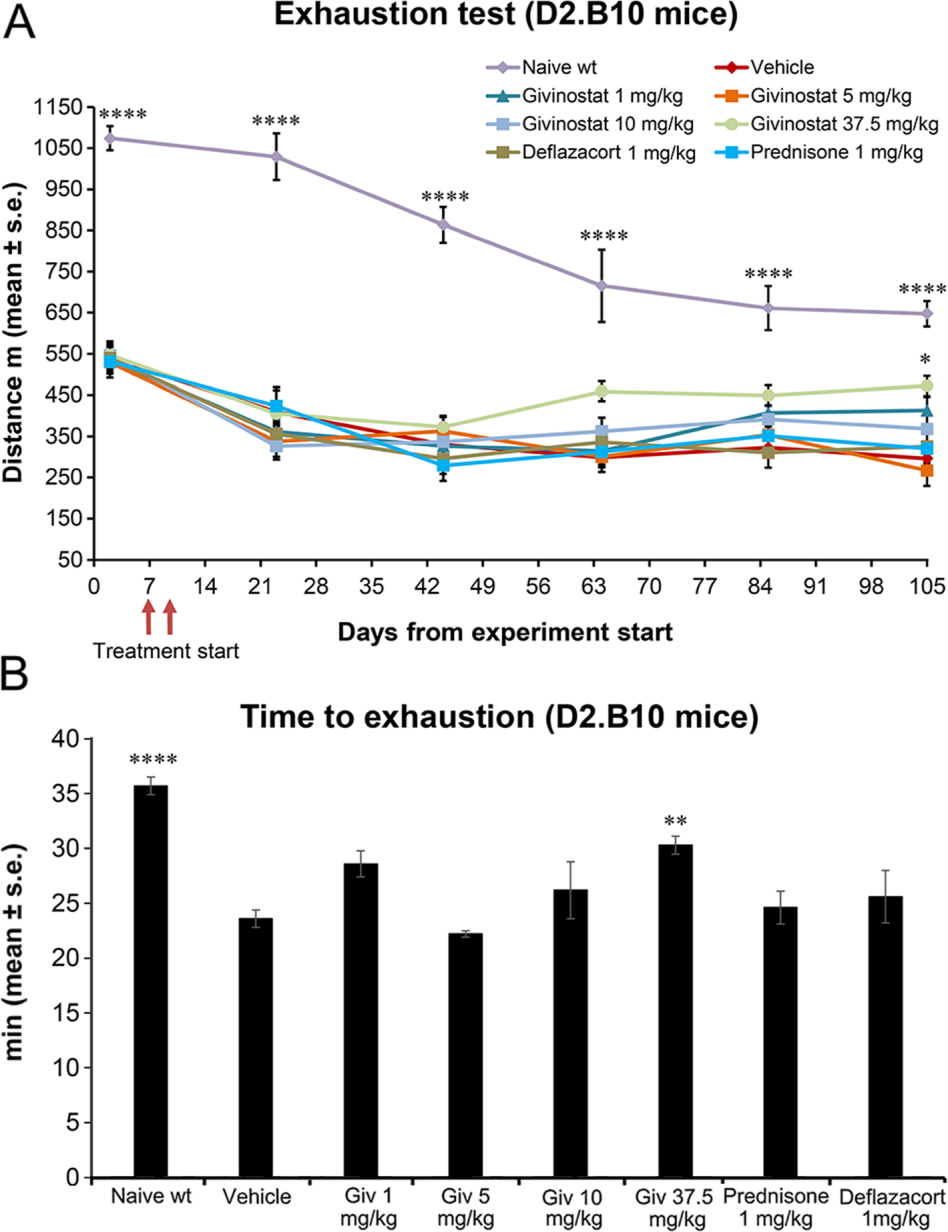
Effect of Givinostat and steroids on both the distance run (**A**) and time to exhaustion at day 105 (**B**) in D2.B10 mice. From day 44, a maximum number of shock (150) was set. Givinostat and steroid treatment started on days 7 and 9, respectively (2-way ANOVA with Bonferroni’s multiple comparison test, *p < 0.05; ****p < 0.0001 vs vehicle; wt: wild type)

During the exhaustion test evaluation, we had to set a maximum number of shocks that mice received until the end of each test with respect to the protocol applied to *mdx* mice in order to avoid unnecessary stress to the animals (see “Assessment of functional tests for the evaluation of treatment efficacy” paragraph in “Materials and methods” section). This adjustment led to an apparent worsening of the performance of healthy DBA/2J which we therefore ascribe to a change in experimental settings and not to an increased exhaustion. Despite the reduced number of shocks, Givinostat at its top dose improved running performance of D2.B10 mice with a clear increase at day 63 (Fig. 8A). This effect lasted until the end of treatment period where the improvement was statistically significant. In fact, on day 105, Givinostat-treated mice were able to run for 473 ± 22 meters, whereas the D2.B10 vehicle-treated mice ran for only 296 ± 17 m (the distance covered by wt mice was 648 ± 31 m). On the contrary, the effect of Deflazacort, prednisone, and of the lower doses of Givinostat was not statistically significant (Fig. 8A).

In addition to the distance, we also analyzed the time to exhaustion at the last time point (day 105). Givinostat, at the highest dose, significantly prolonged the time to exhaustion (Fig. 8B).

### Histopathological analysis

**Fig. 9:**
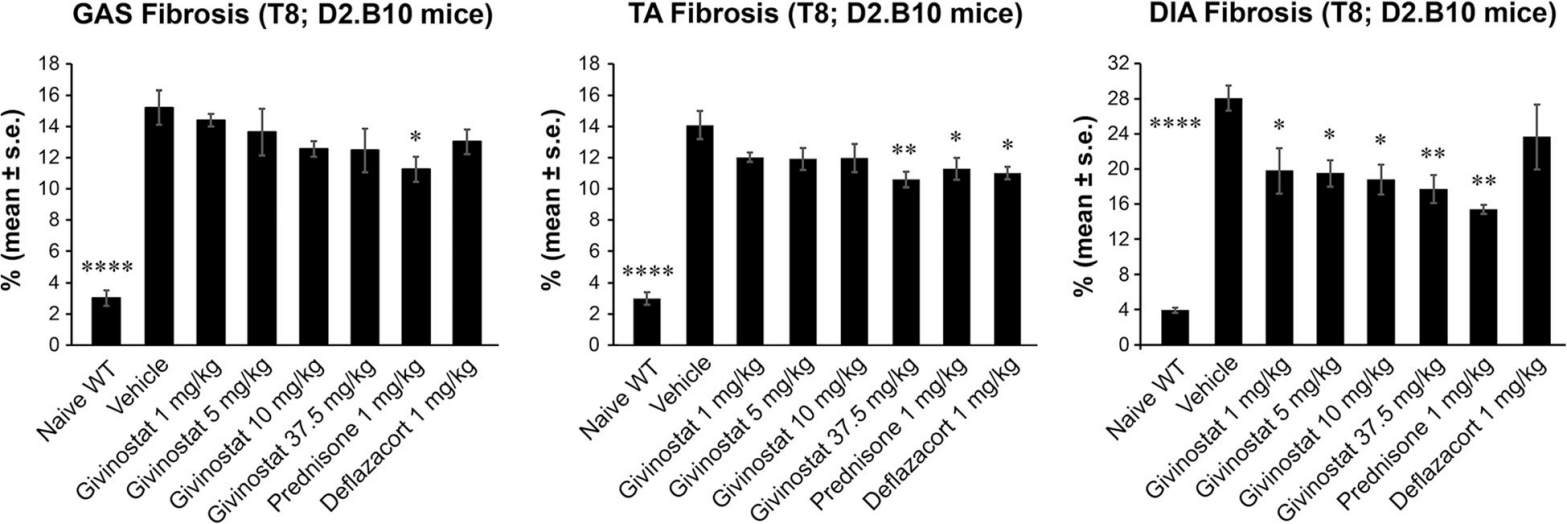
Givinostat and steroid treatment ameliorate muscle fibrosis in D2.B10 mice. Effect of Givinostat and steroids on the percentage of fibrosis in gastrocnemius (GAS), tibialis anterior (TA), and diaphragm (DIA) of D2.B10 mice after 8 weeks of treatment (T8) (1-way ANOVA with Bonferroni’s multiple comparison test, *p < 0.05; **p < 0.01; ****p < 0.0001 vs vehicle; n = 5; wt: wild type)

In DBA/2J wt mice, GAS, TA, and DIA had a low percentage of fibrotic area when compared to dystrophic muscles at both time points T8 (3.0 ± 0.5 %, 3.0 ± 0.4 % and 3.9 ± 0.3 %, respectively) and T16 (1.8 ± 0.5 %, 2.3 ± 0.3 % and 4.7 ± 0.8 %, respectively). In GAS (T8), prednisone significantly reduced fibrosis compared to vehicle-treated mice (by about 26%) (Fig. 9). No significant effect was observed at time point T16 upon either Givinostat or steroid treatment. In TA (T8), both steroids and Givinostat (administered at 37.5 mg/kg) significantly decreased fibrosis compared to vehicle-treated mice (by about 22%) (Fig. 9). No significant effects were observed on fibrosis at time point T16. At T8, DIA of animals treated with Givinostat, as well as with prednisone, showed a significant reduction of the amount of fibrosis by about 33 and 45%, respectively. Deflazacort had no significant effect (Fig. 9). At T16, the efficacy of both Givinostat at 37.5 mg/kg and prednisone was lost, possibly due to the exhaustion of regenerative potential with age [62].

The CSA of GAS and TA of DBA/2J wt mice were higher compared to that of dystrophic mice (1500 μm^2^ at T8 and 900 μm^2^ at T16 in GAS and 1200 μm^2^ at T8 and 1050 μm^2^ at T16 in TA). Moreover, at both T8 and T16, no major changes in CSA distribution were observed in GAS and TA of mice treated with Givinostat administered at 37.5 mg/kg (600 μm^2^ at T8 and 450 μm^2^ at T16 in GAS and 300 μm^2^ at T8 and 300 μm^2^ at T16 in TA) and prednisone at 1 mg/kg (600 μm^2^ at T8 and 300 μm^2^ at T16 in GAS and 300 μm^2^ at T8 and 300 μm^2^ at T16 in TA) compared to D2.B10 vehicle-treated mice (1050 μm^2^ at T8 and 300 μm^2^ at T16 in GAS and 600 μm^2^ at T8 and 300 μm^2^ at T16 in TA).

Nuclear centralization was absent or very low in GAS and TA muscles from wt mice of 24–25 weeks old (around 1%), while it was very diffuse in GAS (25.1 ± 1.5 %) and TA (21.2 ± 1.0%) of D2.B10 vehicle-treated mice of the same age. No marked treatment-related effects were observed: Givinostat at 37.5 mg/kg and prednisone-treated mice sowed a similar nuclear centralization to that of the vehicle group (22.4 ± 2.1 % and 21.6 ± 1.7 %, respectively, in GAS and 19.8 ± 2.4 % and 22.0 ± 2.4 %, respectively in TA).

The histological evaluation of muscle inflammatory infiltrate, adipose tissue deposition, regeneration, and degeneration of the muscular tissue was not significantly different among the treatment groups. In particular, the degree of inflammatory infiltrate and adipose tissue deposition was very limited in all samples and, therefore, not useful for discriminating the efficacy of the different doses of Givinostat or steroids (Additional Table 7).

The statistical analyses of both functional and histological improvements as a function of administered doses are summarized in Additional Table 8 (see Additional Table 8).

## Discussion

DMD patients homozygous for the IAAM Ltbp4 haplotype remained ambulatory significantly longer than those homozygous for the VTTT haplotype [17]. This important correlation was confirmed in other studies [22, 23] where a delay in the loss of ambulation in patients with the IAAM phenotype treated with corticosteroids was also observed.

In our study, we observed that Givinostat was effective in both *mdx* and D2.B10 murine models which functionally represent the mild and more severe phenotypes of DMD, respectively.

It is further interesting to compare the effects of Givinostat on the two mouse strains used in this project. D2.B10 mice could not be chronically dosed by daily gavage and received the drug in their drinking water. Even though water consumption measurements have shown that the total doses that D2.B10 mice received were comparable to those of the *mdx* mice, an accurate PK monitoring upon administration in drinking water is essentially impossible. It is likely however, that the plasma concentration curve of Givinostat will be much flatter with this mode of administration and that lower circulating concentrations will be reached, even though similar AUC values are expected. We notice that in this model, even though strong functional benefit could be shown, only a transient effect on fibrosis and no significant effect on CSAs was observed. It is possible that this lack of effect could relate to the severeness of the disease developed by these animals that may not be efficiently countered by an HDACi. Like for the pharmacologic effect in *mdx* mice, an alternative explanation could be that a robust effect on fibrosis and CSA requires higher circulating concentrations (Cmax-driven effect) that are not attained when the compound is administered with drinking water. In line with this reasoning, Givinostat was inactive in an in vivo pulmonary fibrosis model when administered with the diet (McKinsey TA, unpublished results). This mode of administration is likely to give similar low-level exposures as our administration with drinking water in D2.B10 mice.

Although doubts remain about their ability to prolong ambulation in DMD patients, the pros and cons of their long-term use and the choice of which corticosteroid/regimen to use, GC are routinely used for DMD treatment. We evaluated both prednisone and Deflazacort in the more severe DMD mouse model and we found that there were small differences between the two GC steroids in terms of their ability to improve muscle performance. Prednisone and Deflazacort were unable to affect cross-sectional area or the number of centralized nuclei in D2.B10 muscles. In addition, prednisone significantly reduced fibrosis in TA, GAS, and DIA, while Deflazacort was effective only in TA.

Recently, Givinostat was shown to be highly efficacious in two distinct murine models of diastolic dysfunction with preserved ejection fraction [63]. In these experiments, the drug was admixed to the diet. In these cardiac models, Givinostat blocked left ventricular diastolic dysfunction due to hypertension and suppressed aging-induced diastolic dysfunction in normotensive mice. No effect on fibrosis was observed by the authors, but an impairment of cardiac myofibril relaxation, as a previously unrecognized, myocyte-autonomous mechanism for diastolic dysfunction was discovered that was proposed to be regulated by myofibrillar protein acetylation. We can therefore assert that Givinostat is effective in counteracting fibrosis in the DMD mouse models and not in pulmonary fibrosis and diastolic dysfunction murine models.

A specific role for HDAC2 in the control of myofibrils was suggested that apparently was affected already by very low circulating HDACi concentrations. It would be interesting to investigate if myofiber protein acetylation also occurs in skeletal muscles and to what extent it affects the function of dystrophic muscles.

Givinostat has previously been shown to be effective in the *mdx* mouse model and is presently being studied in phase III clinical trials in DMD patients.

In this preclinical work, we wanted to further dissect the pharmacological activity of the molecule with a special focus on functional readouts, dose dependence, and the influence of Ltbp4 polymorphism. The treadmill assay is known to be associated with large experimental variability. Therefore, the grip test was used for the first time to monitor the functional benefit of Givinostat treatment. In our hands, the data obtained by monitoring forelimb force with this assay, were robust and reproducible and allowed us to establish clean dose-response relationships. Using this readout, Givinostat showed a remarkable, dose-dependent improvement in FNmax with top doses transiently approaching values seen in wt animals. In *mdx* mice, the treadmill distance and time to exhaustion also transiently approached wt values, whereas the analogous data obtained with D2.B10 mice are more difficult to interpret due to protocol changes that were necessary during the experiment to safeguard animal well-being. Still, also in this case, the top dose showed a statistically significant improvement over vehicle-treated animals in both distance and time to exhaustion evaluations. We conclude that Givinostat improves muscle function, irrespective of the Ltbp4 status. Functional benefits were transient in both mouse strains but even in the decline phase a statistically significant difference with respect to vehicle-treated animals was observed.

The mechanism of action of HDACis in muscle regeneration is complex, multifaceted, and not completely understood. Fibro-adipogenic progenitor cells were proposed to be key actors in these processes [56, 57, 62]. In young animals, these cells have been shown to be reprogrammed by HDACis to deliver pro-regenerative factors and to mobilize muscle-resident stem cells to engage in tissue repair [56]. This effect was no longer observable in aged animals, suggesting that exhaustion of regenerative capacity may occur over time [56]. Whether this is the basis for the transient functional improvements observed in our experiments needs to be further explored. Also, the relevance of this exhaustion process for the treatment of human disease with HDACis needs to be established. The outcome of the ongoing clinical trial with Givinostat in adult Becker dystrophy patients (ClinicalTrials.gov identifier: NCT03238235) will be revealing in this respect.

Givinostat, at the top dose, induced significant increases of the CSAs of TA and GAS muscles in *mdx* mice, while no effect on nuclear centralization was seen. Whether the increased CSA is a result of regeneration, of a countering of atrophic processes or due to protection of muscle fibers from damage is presently unknown. We note that Givinostat potently inhibits muscle atrophy pathways in vitro (manuscript in preparation) and has a known anti-inflammatory activity that could mitigate ROS-mediated tissue damage in an inflammatory environment. Both effects could contribute to preserve larger muscle fibers. We also observed a dose-dependent effect on fibrosis in TA, GAS, and DIA muscles from *mdx* mice. The antifibrotic effect was evident at the top doses and this contrasts with the dose dependence of functional improvements that were highly significant also at lower doses. These findings will be further discussed below.

Multiple dose PK experiments were done in *mdx* mice to establish efficacy/PK correlations. To put these data into context, we need to consider that Givinostat is a relatively non-specific inhibitor of all 11 zinc-dependent HDAC subtypes. Minetti and colleagues have shown that Entinostat (MS-275), an HDAC inhibitor that selectively inhibits HDACs 1, 2, and 3 is as active as the pan-HDACi TSA [27], while Colussi and colleagues have demonstrated that ablation of HDAC2 expression recapitulates many features of small molecule inhibitors, confirming a key role of the inhibition of this enzyme in mediating the pharmacologic activity of HDACis [28]. Mechanistically, the impaired nNOS activity in DMD muscles was proposed to lead to a decrease in HDAC2 nitrosylation, causing its retention on miRNA promoters and leading to an increase in the expression of fibrosis/oxidative stress-related genes [61]. miR-206 was suggested to be under the control of HDAC2 and expressed in activated satellite cells where it represses the Pax7 factor, thus allowing differentiation to proceed [64]. Finally, HDAC3 was shown to be an obligate mediator of the inflammatory gene expression program of macrophages [65]. In the light of these findings, it is likely that inhibition of HDACs 1, 2, and 3 is a major driver of the activity of Givinostat in DMD.

From our PK experiments, we conclude that Givinostat rapidly accumulates in muscle tissue, where it reaches a concentration 4.5 times higher than in plasma and that HDAC inhibition is probably going to be transient, since concentration curves rapidly declined after a few hours. Therefore, the improvement of functional and histological parameters obtained in *mdx* mice can be also explained by the high distribution levels of Givinostat into the muscular tissue.

Since the targeted HDACs 1, 2, and 3 are essential enzymes in most cells, their continuous, sustained inhibition would probably not be tolerated. In fact, attempts to deliver HDACis by continuous infusion, leading to their sustained inhibition, were poorly tolerated in our hands (C.S., unpublished observations).

It is interesting to compare our dose-response curves to those reported by Colussi and colleagues using Vorinostat in the *mdx* mouse model [29]. In that report, a bell-shaped dose-response curve of Vorinostat with a peak for both functional and histologic effects at the low dose of 1.2 mg/kg was shown. We notice that this behavior differs from our observations with Givinostat, for which both functional and histologic parameters improved steadily with increasing dose up to 37.5 mg/kg, the highest dose tested. Vorinostat and Givinostat have similar enzyme inhibition profiles [66]. A possible explanation for this discrepancy could be linked to the different administration route of the two compounds: Vorinostat has very poor oral bioavailability in mice and Colussi and colleagues administered this compound i.p., [29] whereas in our experiments Givinostat was given p.o. I.p. dosing is expected to give much higher peak plasma concentrations (Cmax value in CD1 mice was 123 ng/ml at 0.25 h after i.p. administration of 5 mg/kg dose) than p.o. administration. Remarkably, also TSA, when given i.p., is active in the *mdx* model, even though this molecule is extremely short-lived in vivo and was reported to have a half-life of only 6.3 min upon i.p. dosing in mice [67]. However, i.p. dosing of TSA lead to micromolar peak plasma concentrations that are well above the enzyme inhibition IC50 values. Taking these data together, at least some of the pharmacologic effects of HDACis in this model seem to be driven by transient peak plasma concentrations (Cmax values in *mdx* mice treated with Givinostat: 24 ng/ml for the 5 mg/kg p.o. dose; 807 ng/ml for the 37.5 mg/kg p.o. dose).

The question then becomes: how transient target inhibition may still lead to long-lived pharmacodynamic effects? In this respect, an intriguing observation was recently published [57], showing that the pharmacologic effects of HDACi in *mdx* mice are, to a large extent, mediated by extracellular vesicles that are produced by mesenchymal cells such as fibro-adipogenic progenitors (FAPs). These extracellular vesicles are involved in microRNA transfer to muscle stem cells and exposure of dystrophic FAPs to HDACis increases the intra-extracellular vesicles levels of a subset of miRNAs that regulate biological processes such as regeneration, fibrosis, and inflammation. Significantly, extracellular vesicles derived from HDACi-exposed FAPs were able to phenocopy the pharmacologic effects of HDACis upon a single injection in dystrophic mice, indicating long-lasting pharmacodynamic effects.

It is well known that the activity of HDACi on different biological pathways can greatly vary as a function of their concentrations. As an example, we discovered that the anti-inflammatory activity of HDACi is exerted at much lower concentrations compared to those needed for an antitumor activity [68, 69]. Thus, a possible model that reconciles all of these findings could be the following: HDACis may trigger myofiber-autonomous effects leading to functional improvements at relatively low concentrations that can be reached by low-dose administration through oral gavage or upon admixture of the drug to the diet or to drinking water. However, transient exposure to higher concentrations of HDACis, well above the Ki values of the main targets HDACs 1, 2, and 3, may be needed to trigger pro-regenerative and antifibrotic effects. These drug levels can be reached by high-dose p.o. administration or by lower dose i.p. dosing. Transient HDAC inhibition may result in durable pharmacodynamic outcomes that are mediated by extracellular vesicles. This picture needs further confirmation from additional preclinical experiments and quantitative PK/PD correlations need to be established using relevant biomarkers. If confirmed, these data could serve as the basis for optimizing the dosing scheme of HDACis in the clinic.

While the lack of dystrophin that leads to chronic muscle damage is the causal factor of Duchenne dystrophy, the pathologic repair process that is triggered under these conditions and that leads to a progressive substitution of muscle tissue by fibrotic and adipose tissue is a key pathogenic process. Restoring dystrophin expression in all muscle fibers would in theory cure the disease and attempts to at least partially do so using exon skipping oligos or a drug inducing point-mutation read-through have conducted to first approvals of targeted therapies in Duchenne patients [70]. Promising clinical data have been recently published on AAV-based gene therapy, suggesting that further, urgently needed therapy improvements could be in sight for those patients that cannot benefit from presently available therapeutic options [70]. Still, all of these therapeutic approaches have their limitations and even if the present gene therapy approaches were 100% efficacious, they would lead to the expression of a truncated version of dystrophin, at best turning fatal Duchenne into a milder Becker dystrophy type of disease. This implies that a two-pronged approach focusing on both dystrophin restoration and a normalization of the repair process is likely to provide the most benefit to patients.

HDACis are ideally suited for this purpose since they act on multiple aspects of muscle repair through modulation of inflammation, collagen deposition, adipocyte differentiation, and muscle fiber regeneration [71]. In addition, they may have direct effects on muscle contraction. These desirable activities are mediated by different HDAC subtypes that are expressed in different cell types and are likely to have different requirements in terms of HDACi concentration and duration of inhibition. Additional preclinical experiments will be needed to dissect the role of individual HDAC subtypes in these processes and to define the optimal profile and dosing schedule of HDAC inhibitors.

## Conclusion

Our data show that Givinostat improves the functional activities and modifies the histological parameters in a dose-dependent way regardless of the Ltbp4 haplotype; indeed, Givinostat is effective in both the *mdx* (DMD mild phenotype) and in D2.B10 (DMD severe phenotype) murine models.

## Supplementary Information

**Additional file 1: Table 1.** Muscle sampling in both *mdx* and D2.B10 mice. Summary of muscle sampling at the two different time points (T8 = after 8 weeks of treatment; T16 = after 15 weeks of treatment) in both *mdx* and D2.B10 studies (n = 5; GAS = gastrocnemius; TA = tibialis anterior; DIA = diaphragm).

**Additional file 2: Table 2.** Statistical analysis of maximal normalized strength in *mdx* mice. Summary of statistical analysis of maximal normalized strength in wt and *mdx* mice (wt: wild type). 2-way ANOVA with Bonferroni’s multiple comparison test was performed (*p < 0.05; **p < 0.01; ***p < 0.001; ****p < 0.0001 vs Vehicle).

**Additional file 3: Table 3.** Histopathological evaluation of the severity of myodystrophy in different muscles of *mdx* mice at T16. The histopathological method considered some parameters scored by severity and extension of the injury: muscle degeneration/necrosis, regeneration, inflammatory infiltrate, interstitial reaction and adipose tissue deposition. Each parameter was classified by severity (mild = 1, moderate = 2 and severe = 3) and extension (focal = 1, multifocal = 2 and diffuse = 3). The individual severity score was calculated for each animal and an average score per group was determined (group mean total score) (statistical analysis: 1-way ANOVA with Bonferroni’s multiple comparison test. Mean values ± SD vs Vehicle; n = 5; T16 = sampling after 15 weeks of treatment; DIA = diaphragm; GAS = gastrocnemius; MTS = mean total score; TA = tibialis anterior; wt = wild type;).

**Additional file 4: Table 4.** Summary of the statistical analysis results of functional and histological parameters in *mdx* mice. Givinostat administered at the dose of 37.5 mg/kg led to significant improvements in both functional tests (T8 and T16) and histological evaluations (except for heart) (T16). Statistical analysis: functional tests, 2-way ANOVA with Bonferroni’s multiple comparison test; histological parameters, 1-way ANOVA with Bonferroni’s multiple comparison test. Mean values ± s.e. (*p < 0.05; **p < 0.01; ***p < 0.001; ****p < 0.0001 vs Vehicle; ns = not significant; s = significant based on multiplicative model effect in gastrocnemius and additive model effect in tibialis anterior, as described in *Statistical analysis* section in Materials and Methods paragraph; T8 = sampling after 8 weeks of treatment; T16 = sampling after 15 weeks of treatment; CSA = cross sectional area).

**Additional file 5: Table 5.** Summary of differentially expressed miRNA in Naive wt, Naive mdx, Givinostat 37.5 mg/kg and vehicle *mdx* mice. A large number of statistically significant, differentially expressed miRNAs could be identified in all contrasts. Included: number of miRNAs used in the analysis with non-zero total read count; up: number of miRNAs upregulated at FDR < 0.05; down: number of miRNAs downregulated at FDR < 0.05. FDR: false discovery rate.

**Additional file 6: Table 6.** Statistical analysis of maximal normalized strength in D2.B10 mice. Summary of statistical analysis of maximal normalized strength in wt and D2.B10 mice (wt: wild type). 2-way ANOVA with Bonferroni’s multiple comparison test was performed (*p < 0.05; **p < 0.01; ***p < 0.001; ****p < 0.0001 vs Vehicle).

**Additional file 7: Table 7.** Histopathological evaluation of the severity of myodystrophy in different muscles of D2.B10 mice at T8 and T16. The histopathological method considered some parameters scored by severity and extension of the injury: muscle degeneration/necrosis, regeneration, inflammatory infiltrate, interstitial reaction and adipose tissue deposition. Each parameter was classified by severity (mild = 1, moderate = 2 and severe = 3) and extension (focal = 1, multifocal = 2 and diffuse = 3). The individual severity score was calculated for each animal and an average score per group was determined (group mean total score) (statistical analysis: 1-way ANOVA with Bonferroni’s multiple comparison test. Mean values ± SD vs Vehicle; n = 5; T8 = sampling after 8 weeks of treatment; T16 = sampling after 15 weeks of treatment; DIA = diaphragm; GAS = gastrocnemius; MTS = mean total score; TA = tibialis anterior; wt = wild type;).

**Additional file 8: Table 8.** Summary of the statistical analysis results of functional and histological parameters in D2.B10 mice. Givinostat administered at the doses of 5, 10 or 37.5 mg/kg and steroids (except for Deflazacort at T16) led to significant improvements in grip strength test, whereas, we observed a statistically significant improvement in the exhaustion test only with the highest dose of Givinostat at T16. For the histological analysis we observed a statistically significant result only in fibrosis at T8 in diaphragm (DIA) for all the doses of Givinostat administered and for Prednisone treatment. Givinostat at 37.5 mg/kg also significantly counteracted fibrosis in tibialis anterior (TA); Prednisone was able to diminish fibrosis in TA and gastrocnemius (GAS), whereas the antifibrotic effect of Deflazacort was observed only in TA. Statistical analysis: functional tests, 2-way ANOVA with Bonferroni’s multiple comparison test; histological parameters, 1-way ANOVA with Bonferroni’s multiple comparison test. Mean values ± s.e. (*p < 0.05; **p < 0.01; ***p < 0.001; ****p < 0.0001 vs Vehicle; ns = not significant; T8 = sampling after 8 weeks of treatment; T16 = sampling after 15 weeks of treatment; CSA = cross sectional area).

**Additional file 9: Figure 1.** Experimental plan of treatments and functional tests in *mdx* (A) and D2.B10 (B) studies. Givinostat was orally administered by daily gavage in *mdx* mice, whereas it was dissolved in drinking water for D2.B10 mice starting from day 7. Deflazacort and Prednisone were weekly administered by i.p. injection at the dose of 1 mg/kg starting from day 9 only in D2.B10 mice. The grip strength and run to exhaustion tests have been conducted after a training period of 3 and 4 days, respectively, during which mice become familiar with the procedures. Grip strength test was performed every week in both the studies, instead, run to exhaustion performance of *mdx* and D2.B10 mice was evaluated every 14 or 21 days, respectively, using a treadmill apparatus.

**Additional file 10: Figure 2.** Effect of Givinostat on *mdx* mice body weight. Baseline BW values of 8 weeks old C57BL/10J wt and Naive *mdx* mice were 24.8 ± 0.8 and 27.7 ± 0.8 grams, respectively. At the end of the treatment period (day 105), the BW of mice treated with 25 and 37.5 mg/kg of Givinostat was 32.9 ± 0.9 and 33.1 ± 0.9 g respectively, i.e. similar to that of the wt mice (33.3 ± 0.9 g) but significantly different (*p* < 0.01 and *p* < 0.05, respectively) from that of the vehicle-treated *mdx* mice (37.2 ± 0.8 g), suggesting that Givinostat counteracted the pathologic BW gain of *mdx* mice (BW = body weight; wt = wild type). 2-way ANOVA with Bonferroni’s multiple comparison test was performed.

**Additional file 11: Figure 3.** Effect of Givinostat, Prednisone and Deflazacort on D2.B10 mice body weight. The baseline BW mean value of D2.B10 mice was 18.2 ± 0.6 grams, whereas the weight of the wt mice was 24.8 ± 0.5 grams (*p* < 0.0001) and they still remained heavier than the dystrophic mice for all the duration of the experiment: at day 105, the mean BW of D2.B10 mice was 23.3 ± 0.6 g, whereas the BW of wt mice was 29.4 ± 0.5 g (*p* < 0.0001). There were no significant differences in BW of D2.B10 vehicle-treated mice compared to that of D2.B10 mice treated with either Givinostat or steroids (BW = body weight; wt = wild type). 2-way ANOVA with Bonferroni’s multiple comparison test was performed.

## Data Availability

The datasets used and/or analyzed during the current study are available from the corresponding author on reasonable request.

## Abbreviations

BW: Body weight
CSA: Cross-sectional area
DMD: Duchenne muscular dystrophy
DIA: Diaphragm
FNmax: Maximal normalized strength
GAS: Gastrocnemius
GC: Glucorticoids
HDAC: Histone deacetylase
HDACi: Histone deacetylase inhibitor
*LTBP4* gene: Latent transforming growth factor-β binding protein 4
miRNAs: MicroRNAs
ns: Not significant
PK: Pharmacokinetic
TA: Tibialis anterior
TGF-β: Transforming growth factor-β
wt: Wild type
