# Supplementary material for: The pan HDAC inhibitor Givinostat improves muscle function and histological parameters in two Duchenne muscular dystrophy murine models expressing different haplotypes of the LTBP4 gene
Source: Skelet Muscle. 2021 Jul 22;11:19. doi: 10.1186/s13395-021-00273-6 (PMC8296708; doi:10.1186/s13395-021-00273-6)
Supplement: Supplementary file 1 — Additional file 1: Table 1. Muscle sampling in both mdx and D2.B10 mice. Summary of muscle sampling at the two different time points (T8 = after 8 weeks of treatment; T16 = after 15 weeks of treatment) in both mdx and D2.B10 studies (n = 5; GAS = gastrocnemius; TA = tibialis anterior; DIA = diaphragm). [file 13395_2021_273_MOESM1_ESM.docx]

**Additional Table 1**
