# Supplementary material for: The pan HDAC inhibitor Givinostat improves muscle function and histological parameters in two Duchenne muscular dystrophy murine models expressing different haplotypes of the LTBP4 gene
Source: Skelet Muscle. 2021 Jul 22;11:19. doi: 10.1186/s13395-021-00273-6 (PMC8296708; doi:10.1186/s13395-021-00273-6)
Supplement: Supplementary file 4 — Additional file 4: Table 4. Summary of the statistical analysis results of functional and histological parameters in mdx mice. Givinostat administered at the dose of 37.5 mg/kg led to significant improvements in both functional tests (T8 and T16) and histological evaluations (except for heart) (T16). Statistical analysis: functional tests, 2-way ANOVA with Bonferroni’s multiple comparison test; histological parameters, 1-way ANOVA with Bonferroni’s multiple comparison test. Mean values ± s.e. (*p < 0.05; **p < 0.01; ***p < 0.001; ****p < 0.0001 vs Vehicle; ns = not significant; s = significant based on multiplicative model effect in gastrocnemius and additive model effect in tibialis anterior, as described in Statistical analysis section in Materials and Methods paragraph; T8 = sampling after 8 weeks of treatment; T16 = sampling after 15 weeks of treatment; CSA = cross sectional area). [file 13395_2021_273_MOESM4_ESM.docx]

**Additional Table 4**
