# Supplementary material for: The pan HDAC inhibitor Givinostat improves muscle function and histological parameters in two Duchenne muscular dystrophy murine models expressing different haplotypes of the LTBP4 gene
Source: Skelet Muscle. 2021 Jul 22;11:19. doi: 10.1186/s13395-021-00273-6 (PMC8296708; doi:10.1186/s13395-021-00273-6)
Supplement: Supplementary file 5 — Additional file 5: Table 5. Summary of differentially expressed miRNA in Naive wt, Naive mdx, Givinostat 37.5 mg/kg and vehicle mdx mice. A large number of statistically significant, differentially expressed miRNAs could be identified in all contrasts. Included: number of miRNAs used in the analysis with non-zero total read count; up: number of miRNAs upregulated at FDR < 0.05; down: number of miRNAs downregulated at FDR < 0.05. FDR: false discovery rate. [file 13395_2021_273_MOESM5_ESM.docx]

**Additional Table 5**
