# Supplementary material for: The pan HDAC inhibitor Givinostat improves muscle function and histological parameters in two Duchenne muscular dystrophy murine models expressing different haplotypes of the LTBP4 gene
Source: Skelet Muscle. 2021 Jul 22;11:19. doi: 10.1186/s13395-021-00273-6 (PMC8296708; doi:10.1186/s13395-021-00273-6)
Supplement: Supplementary file 6 — Additional file 6: Table 6. Statistical analysis of maximal normalized strength in D2.B10 mice. Summary of statistical analysis of maximal normalized strength in wt and D2.B10 mice (wt: wild type). 2-way ANOVA with Bonferroni’s multiple comparison test was performed (*p < 0.05; **p < 0.01; ***p < 0.001; ****p < 0.0001 vs Vehicle). [file 13395_2021_273_MOESM6_ESM.docx]

**Additional Table 6**
