# Supplementary material for: The pan HDAC inhibitor Givinostat improves muscle function and histological parameters in two Duchenne muscular dystrophy murine models expressing different haplotypes of the LTBP4 gene
Source: Skelet Muscle. 2021 Jul 22;11:19. doi: 10.1186/s13395-021-00273-6 (PMC8296708; doi:10.1186/s13395-021-00273-6)
Supplement: Supplementary file 7 — Additional file 7: Table 7. Histopathological evaluation of the severity of myodystrophy in different muscles of D2.B10 mice at T8 and T16. The histopathological method considered some parameters scored by severity and extension of the injury: muscle degeneration/necrosis, regeneration, inflammatory infiltrate, interstitial reaction and adipose tissue deposition. Each parameter was classified by severity (mild = 1, moderate = 2 and severe = 3) and extension (focal = 1, multifocal = 2 and diffuse = 3). The individual severity score was calculated for each animal and an average score per group was determined (group mean total score) (statistical analysis: 1-way ANOVA with Bonferroni’s multiple comparison test. Mean values ± SD vs Vehicle; n = 5; T8 = sampling after 8 weeks of treatment; T16 = sampling after 15 weeks of treatment; DIA = diaphragm; GAS = gastrocnemius; MTS = mean total score; TA = tibialis anterior; wt = wild type;). [file 13395_2021_273_MOESM7_ESM.docx]

Additional Table 7
