# Supplementary material for: The pan HDAC inhibitor Givinostat improves muscle function and histological parameters in two Duchenne muscular dystrophy murine models expressing different haplotypes of the LTBP4 gene
Source: Skelet Muscle. 2021 Jul 22;11:19. doi: 10.1186/s13395-021-00273-6 (PMC8296708; doi:10.1186/s13395-021-00273-6)
Supplement: Supplementary file 8 — Additional file 8: Table 8. Summary of the statistical analysis results of functional and histological parameters in D2.B10 mice. Givinostat administered at the doses of 5, 10 or 37.5 mg/kg and steroids (except for Deflazacort at T16) led to significant improvements in grip strength test, whereas, we observed a statistically significant improvement in the exhaustion test only with the highest dose of Givinostat at T16. For the histological analysis we observed a statistically significant result only in fibrosis at T8 in diaphragm (DIA) for all the doses of Givinostat administered and for Prednisone treatment. Givinostat at 37.5 mg/kg also significantly counteracted fibrosis in tibialis anterior (TA); Prednisone was able to diminish fibrosis in TA and gastrocnemius (GAS), whereas the antifibrotic effect of Deflazacort was observed only in TA. Statistical analysis: functional tests, 2-way ANOVA with Bonferroni’s multiple comparison test; histological parameters, 1-way ANOVA with Bonferroni’s multiple comparison test. Mean values ± s.e. (*p < 0.05; **p < 0.01; ***p < 0.001; ****p < 0.0001 vs Vehicle; ns = not significant; T8 = sampling after 8 weeks of treatment; T16 = sampling after 15 weeks of treatment; CSA = cross sectional area). [file 13395_2021_273_MOESM8_ESM.docx]

**Additional Table 8**
