# Supplementary figures and images for: The pan HDAC inhibitor Givinostat improves muscle function and histological parameters in two Duchenne muscular dystrophy murine models expressing different haplotypes of the LTBP4 gene
Source: Skelet Muscle. 2021 Jul 22;11:19. doi: 10.1186/s13395-021-00273-6 (PMC8296708; doi:10.1186/s13395-021-00273-6)

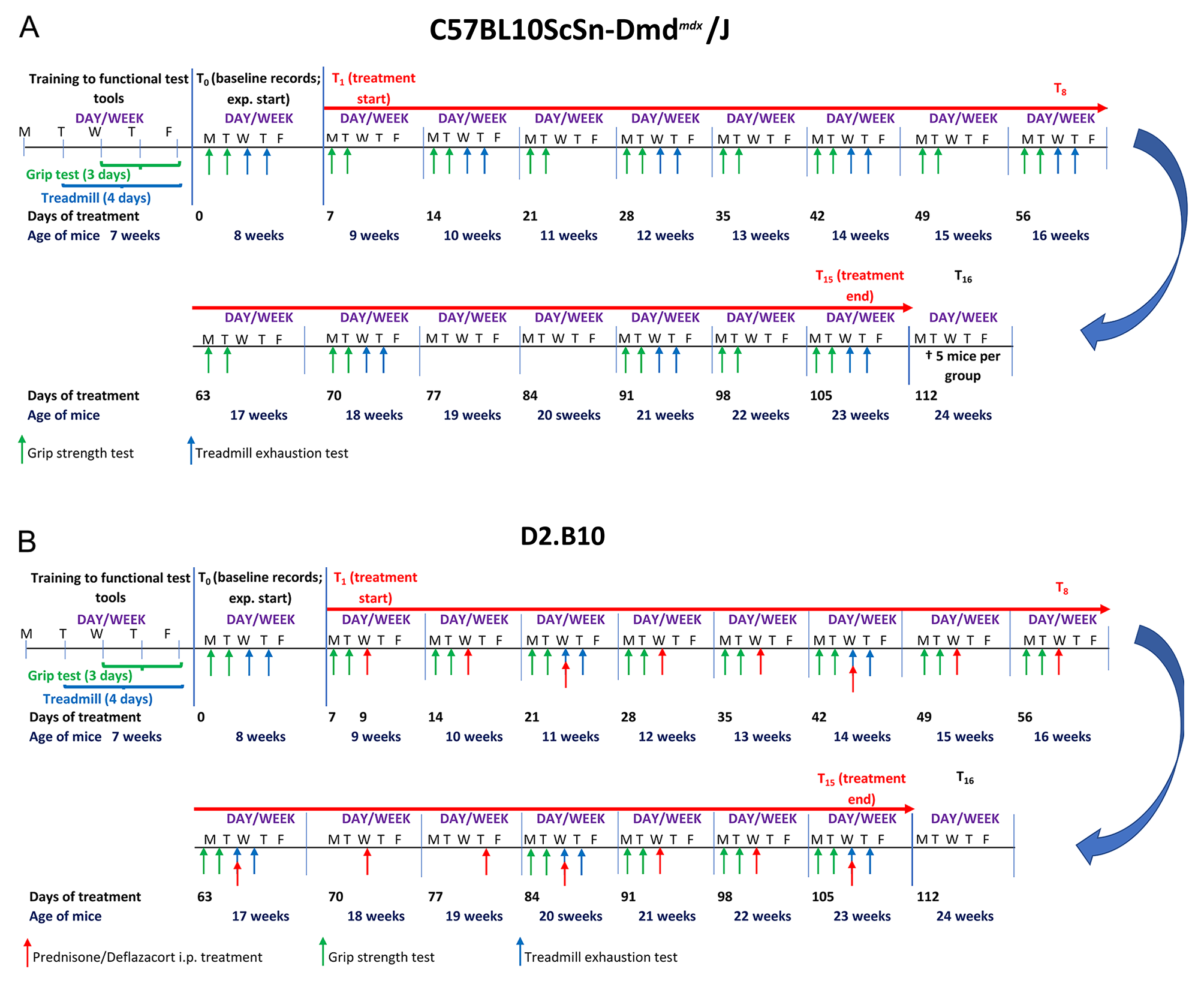

Supplement: Supplementary file 9 — Additional file 9: Figure 1. Experimental plan of treatments and functional tests in mdx (A) and D2.B10 (B) studies. Givinostat was orally administered by daily gavage in mdx mice, whereas it was dissolved in drinking water for D2.B10 mice starting from day 7. Deflazacort and Prednisone were weekly administered by i.p. injection at the dose of 1 mg/kg starting from day 9 only in D2.B10 mice. The grip strength and run to exhaustion tests have been conducted after a training period of 3 and 4 days, respectively, during which mice become familiar with the procedures. Grip strength test was performed every week in both the studies, instead, run to exhaustion performance of mdx and D2.B10 mice was evaluated every 14 or 21 days, respectively, using a treadmill apparatus. [file 13395_2021_273_MOESM9_ESM.tif]

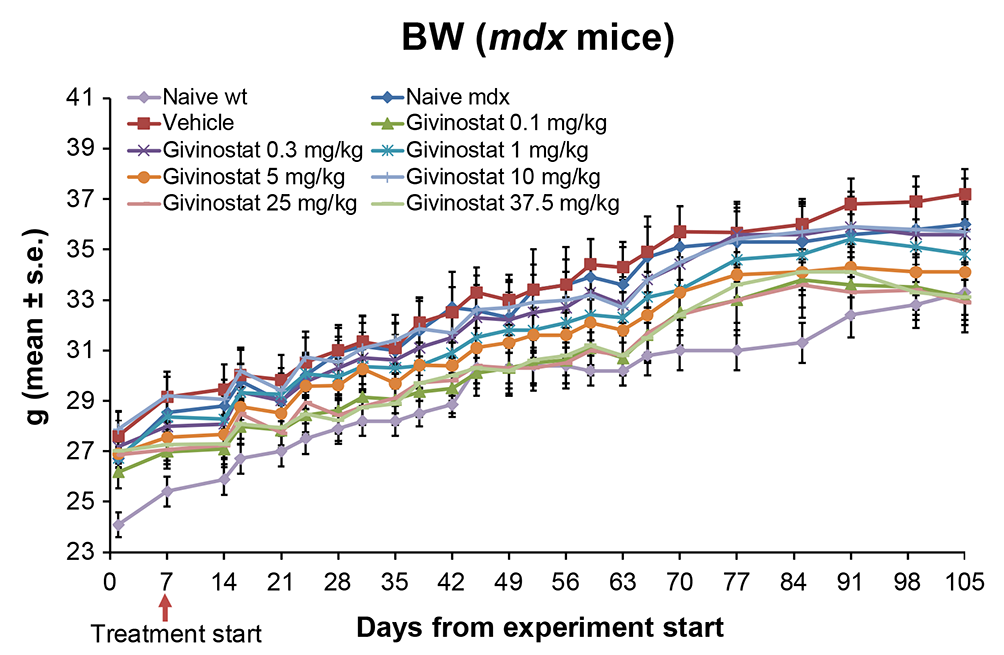

Supplement: Supplementary file 10 — Additional file 10: Figure 2. Effect of Givinostat on mdx mice body weight. Baseline BW values of 8 weeks old C57BL/10J wt and Naive mdx mice were 24.8 ± 0.8 and 27.7 ± 0.8 grams, respectively. At the end of the treatment period (day 105), the BW of mice treated with 25 and 37.5 mg/kg of Givinostat was 32.9 ± 0.9 and 33.1 ± 0.9 g respectively, i.e. similar to that of the wt mice (33.3 ± 0.9 g) but significantly different (p < 0.01 and p < 0.05, respectively) from that of the vehicle-treated mdx mice (37.2 ± 0.8 g), suggesting that Givinostat counteracted the pathologic BW gain of mdx mice (BW = body weight; wt = wild type). 2-way ANOVA with Bonferroni’s multiple comparison test was performed. [file 13395_2021_273_MOESM10_ESM.tif]

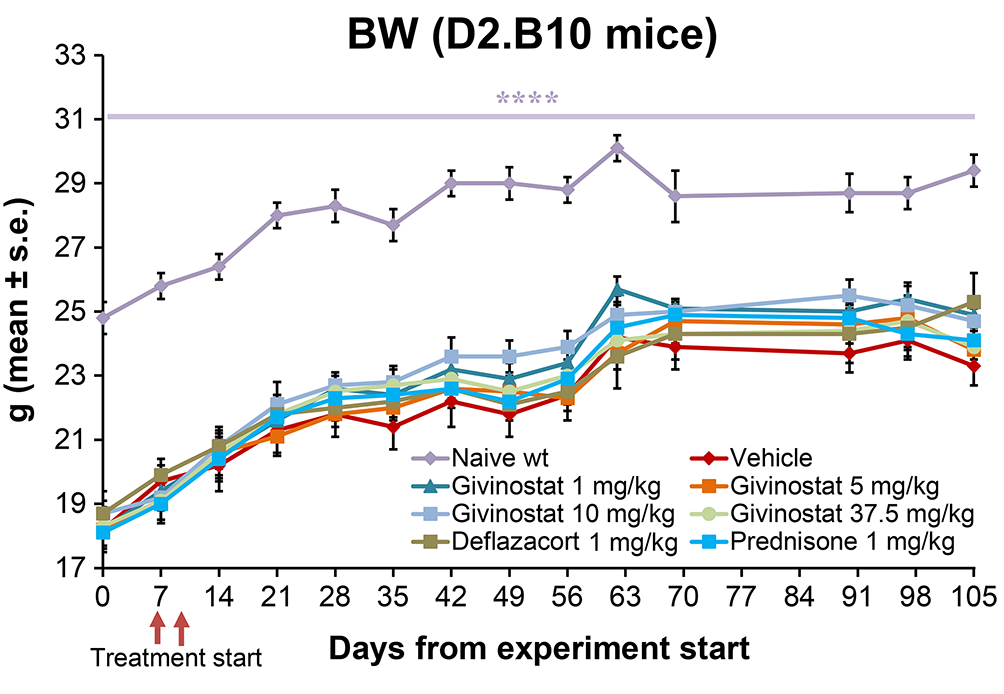

Supplement: Supplementary file 11 — Additional file 11: Figure 3. Effect of Givinostat, Prednisone and Deflazacort on D2.B10 mice body weight. The baseline BW mean value of D2.B10 mice was 18.2 ± 0.6 grams, whereas the weight of the wt mice was 24.8 ± 0.5 grams (p < 0.0001) and they still remained heavier than the dystrophic mice for all the duration of the experiment: at day 105, the mean BW of D2.B10 mice was 23.3 ± 0.6 g, whereas the BW of wt mice was 29.4 ± 0.5 g (p < 0.0001). There were no significant differences in BW of D2.B10 vehicle-treated mice compared to that of D2.B10 mice treated with either Givinostat or steroids (BW = body weight; wt = wild type). 2-way ANOVA with Bonferroni’s multiple comparison test was performed. [file 13395_2021_273_MOESM11_ESM.tif]
